# Efficacy and Safety of Fire Needle Therapy for Flat Warts: Evidence from 29 Randomized Controlled Trials

**DOI:** 10.1155/2021/9513762

**Published:** 2021-01-16

**Authors:** Ying Zhang, Jing-Si Jiang, Le Kuai, Yue Luo, Jia-Le Chen, Yan-Jiao Wang, Rong Xu, Meng Xing, Liu Liu, Xin Li, Bin Li

**Affiliations:** ^1^Department of Dermatology, Yueyang Hospital of Integrated Traditional Chinese and Western Medicine, Shanghai University of Traditional Chinese Medicine, Shanghai 200437, China; ^2^Institute of Dermatology, Shanghai Academy of Traditional Chinese Medicine, Shanghai 201203, China; ^3^Shanghai Dermatology Hospital, Tongji University, Shanghai 200443, China

## Abstract

Flat warts are a common and recurrent skin disease that has no specific antiviral treatment. As an alternative or complementary therapy, fire needle therapy has been widely used in the treatment of flat warts. The objective of this study was to systematically evaluate the efficacy and safety of fire needle therapy for flat warts. Using the search terms “flat warts” and “fire needle,” we searched the PubMed, Embase, Cochrane, China National Knowledge Infrastructure, Wanfang Data Knowledge Service Platform, Chinese biomedical (SinoMed) database, and the China Science and Technology Journal databases for studies until March 12, 2020. Randomized controlled trials comparing fire needle therapies with conventional therapies were also included. We calculated the risk ratios (RR) and mean differences with a 95% confidence interval (CI). We analyzed 29 trials involving 2,666 patients. Results showed that the use of fire needle therapy alone may have a higher efficacy rate compared with that of an immunomodulator (RR = 1.11, 95% CI: 1.03 to 1.20, *I*^2^ = 0%, *P* = 0.006; RR = 1.19, 95% CI: 1.03 to 1.37, *I*^2^ = 70%, *P* = 0.02, respectively) or tretinoin (RR = 1.39, 95% CI: 1.25 to 1.55, *I*^2^ = 0%, *P* < 0.00001), with a lower risk of blisters (*P* = 0.03) or erythema (*P* = 0.04), but with a higher risk of pigmentation (*P* = 0.02). We also determined the efficacy rate of fire needle therapy in combination with traditional Chinese medicine (RR = 1.16, 95% CI: 1.10 to 1.23, *I*^2^ = 21%, *P* < 0.00001), immunomodulators (RR = 1.17, 95% CI: 1.07 to 1.28, *I*^*2*^ = 33%, *P* = 0.0005), imiquimod (RR = 1.21, 95% CI: 1.04 to 1.42, *P* = 0.02), or as multidrug therapies (RR = 1.15, 95% CI: 1.07 to 1.24, *I*^*2*^ = 0%, *P* = 0.0001) and found that the combination treatments could reduce recurrence rates (*P* < 0.00001) and provided a lower risk of desquamation (*P* = 0.006). In conclusion, fire needle therapy seems to be effective for flat warts, with a reduced incidence of adverse events, such as blisters, erythema, and desquamation, but may increase incidence of pigmentation.

## 1. Introduction

Flat warts, which appear as flat, light brown papules, are caused by human papillomavirus (HPV) infection, especially types 3/10 and 28 [1]. Epidemiological surveys have shown that the incidence of flat warts has reached 1.77% in recent years, accounting for 11.4% of facial skin diseases, mainly occurring in children and adolescents [2]. Although 65% to 78% of skin warts may resolve within 2 years, skin warts in adults rarely repair themselves and usually last 5 to 10 years [3, 4], affecting general appearance and mental health, and cause major psychosocial problems.

The stratum corneum of the flat wart lesions in the basal layer of the skin was observed to have epidermal thickening and hyperkeratinization [5]. Histopathological analysis showed that many vacuole-like clear cells were found in the upper and granular layers of the spinous layer [6]. The diagnosis of flat warts is usually based on clinical symptoms: apical papules, minimal scale, and a slight elevation of 2 to 4 mm in diameter of the papules [7].

The mechanisms linking HPV to flat warts have not yet been identified, but it is generally believed that flat warts are closely related to changes in the human immune system [8]. There is no antiviral treatment specific to HPV; however, cell-mediated immunity against viruses has been reported to have a significant effect on flat warts [9]. Current treatments for flat warts, including salicylic acid, cryotherapy, bleomycin, 5-fluorouracil, and lasers, destroy the wart body, correct abnormal proliferation, and differentiation, and stimulate the local or systemic immune response [10–15]. Although these therapies have been proven effective, treating the adverse effects of warts, including infection, blisters, or scars, requires the development of a formulation with similar therapeutic effects but fewer adverse events [8, 16].

In China, fire needle therapy, a type of acupuncture therapy, has been used to treat skin diseases. It is an external treatment method that uses a specific needle that is heated until it burns red and is quickly stabbed into diseased local lesions or acupuncture points. It could stimulate and dredge the meridians and accelerate the flow of Qi and blood thus dissipating nodules. On this basis, the fire needles therapy has been proven to treat nodular prurigo [17], moderate severe acne [18], vitiligo [19]and psoriasis [20, 21]. Recently, fire needle therapy has also been used to treat flat warts, and its possible therapeutic mechanism may be destroying the wart body, improving local circulation, and promoting the local immune response. Additionally, the 2014 British Dermatology Association guidelines recommended acupuncture as a treatment for flat warts on the hands or face [8], and the Chinese fire needle guidelines also recommend acupuncture for flat warts [22].

Nevertheless, there is still a lack of systematic reviews comparing the use of fire needle therapy combined with different medications for flat warts. Here, we conducted a systematic review of randomized controlled trials (RCTs) to evaluate the efficacy and safety of fire needle therapy for flat warts.

## 2. Materials and Methods

### 2.1. Materials and Methods for Fire Needle Therapy

**Figure 1:**
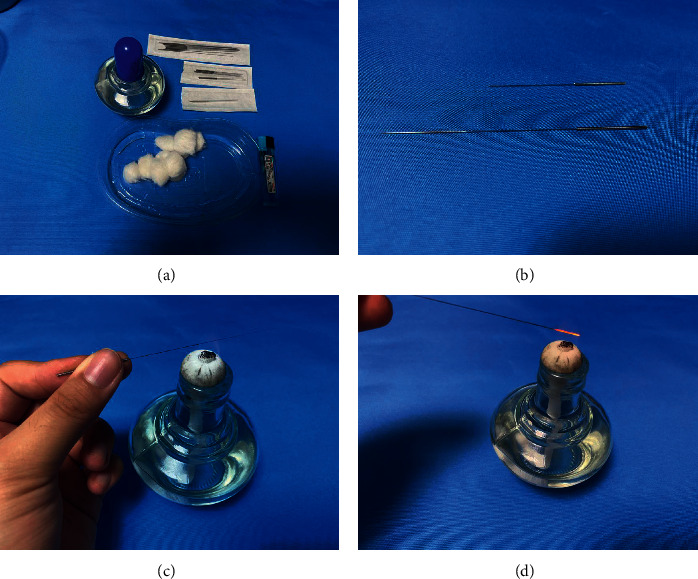
Materials and methods of preparing the fire needle (a–d).

After informing the patient of the procedure, a surgeon used 75% alcohol to disinfect his hands and the acupuncture site of the patient. The operation included the following steps:

1. An alcohol lamp and a disposable sterile fire needle were prepared (Figures 1(a) and 1(b)).
2. The alcohol lamp was lit and was moved continuously from the needle root, along the needle body, to the needle tip (Figure 1(c)).
3. The needle tip and the front of the needle body were heated over the outer flame, and the needle body was moved over the flame until it turned red (Figure 1(d)).
4. The center of the wart body was quickly punctured vertically, and the needle was withdrawn. Small warts only needed to be pricked using one needle; large warts were punctured around the lesion with multiple needles, and the puncture depth did not exceed the base of the lesion.

### 2.2. Registration

This systematic review was reported following the Preferred Reporting Items for Systematic Reviews and Meta-Analyses (PRISMA) guidelines (Supplementary file 1: Table S1) [23]. The PROSPERO registration number of this study is CRD42020185678.

### 2.3. Search Trials

Two reviewers (Le Kuai and Yue Luo) searched for relevant RCTs published in the following databases: PubMed, Embase, Cochrane, China National Knowledge Infrastructure (CNKI), Wanfang Medical Database, Chinese biomedical (SinoMed) database, and the China Science and Technology Journal Database (VIP), from their inception to March 12, 2020. Furthermore, the clinicaltrials.gov and the China Clinical Trial registry website were thoroughly searched to confirm the availability of relevant unpublished studies. Studies were restricted to the English and Chinese languages. The search strategy is listed in Supplementary File 2: Table S2. A total of 341 articles were retrieved.

### 2.4. Study Selection

We screened the titles, abstracts, and full texts of these 341 trials using the following inclusion criteria: participants, inclusion of patients diagnosed with flat warts, regardless of the age, sex, and disease duration; intervention, fire needle, or combined therapies as the intervention; comparison, control groups of conventional therapies; outcome, standardized therapeutic evaluation (efficacy rate) as the outcome; study design, RCTs. A total of 312 trials were excluded by the following exclusion criteria: case reports, reviews, animal studies, and studies containing fire needle therapy or combined therapies for a control group. The inclusion and exclusion of studies were formulated according to participants, intervention, comparison, outcome, and study design (PICOS) principle (Supplementary File 3: Table S3).

### 2.5. Data Extraction

Two investigators independently scrutinized the full texts of the selected studies. Two authors (Jia-le Chen and Yan-jiao Wang) completed the self-designed data extraction form (Table 1), including the general information (i.e., the first author, study design, and year of publication), participant characteristics (i.e., average age, sample size, and disease duration), diagnostic criteria, interventions, duration of treatments, primary or secondary outcomes, adverse events, and recurrence rates.

### 2.6. Risk of Bias Assessment

Two authors (Meng Xing and Rong Xu) independently conducted risk assessments, using the Cochrane bias risk tool [24]. The evaluation items included random sequence generation (selection bias), allocation concealment (selection bias), blinding of participants and personnel (implementation bias), blinding of result evaluation (monitoring bias), incomplete result data (wear bias), selective reporting (reporting bias), and other biases. We assessed the risk of bias by using the terms “low risk,” “unclear risk,” and “high risk.” When disagreements occurred, the two authors had discussions to address these issues. If differences still existed, a third author, Bin Li, was invited to make a final decision.

### 2.7. Level of Evidence

The level of evidence combines considerations of risk of bias, directness, heterogeneity, precision, and publication bias classified into grades of recommendation, assessment, development, and evaluation working (GRADE) criteria: very low-quality evidence (+), low-quality evidence (++), moderate-quality evidence (+++), and high-quality evidence (++++). The GRADEpro guideline development tool (GDT) platform (https://gradepro.org) was adopted to create a summary of findings tables for Cochrane systematic reviews and assess the level of evidence of the outcomes.

### 2.8. Statistical Analysis

In this meta-analysis, we used RevMan 5.3 software (version 5.3, Cochrane Collaboration) to calculate the risk ratios (RR) and the mean differences (MD) with a 95% confidence interval (CI). Standard mean differences (SMD) were used when the measurement criteria were not the same. Heterogeneity was tested using the *I*^2^ statistic. A fixed effect model was used when *P* > 0.1 and *I*^2^ < 50%; otherwise, when *I*^2^ > 50%, subgroup analysis was adopted to resolve methodological and clinical heterogeneity. When there was heterogeneity that could not be readily explained, a random effect model was considered. We performed a sensitivity analysis of all indices to test the stability of the results when necessary. Since studies with negative results could remain unpublished, a funnel plot was used to analyze publication bias across the studies.

### 2.9. Outcomes

According to the PICOS principle, we use efficiency rate as an indicator that emphasizes the primary outcome of patients and include secondary outcomes. The primary outcome was divided into the following four categories: cured, defined by complete resolution of the skin lesions; significantly effective, defined by partial resolution of the skin lesions or if the skin lesion scores were ≥70% but <100%; effective, defined by partial resolution of the skin lesions or if the skin lesion scores were ≥30% but <70%; and ineffective, defined by an insignificant resolution of the skin lesions or if the skin lesion scores were <30% [25]. We used the following formula to calculate the total effective rate: total effective rate = (number of “cured” patients + number of “significantly effective” patients + number of “effective” patients)/total number of patients × 100%. The secondary outcomes were skin lesion scores, cytokine levels, Dermatology Life Quality Index (DLQI), recurrence rate, and adverse events.

## 3. Results

### 3.1. Included Studies and Their Characteristics

**Figure 2:**
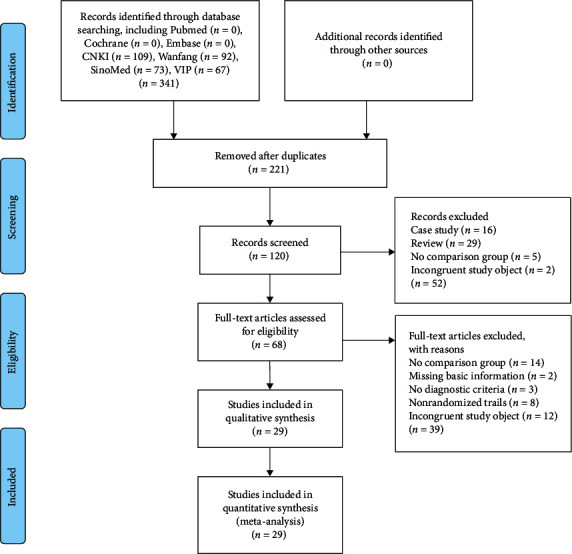
Flowchart of the search strategy and study selection, according to the Preferred Reporting Items for Systematic Reviews and Meta-Analyses (PRISMA) guidelines. CNKI: Chinese National Knowledge Infrastructure database, Wanfang: Wanfang Data Knowledge Service Platform, SinoMed: Chinese Biomedical Database, VIP: China Science and Technology Journal Database, and CBM: Chinese Biomedicine Database.

We obtained 341 relevant studies from seven databases; after the removal of 221 duplicate reports, 120 reports remained. After title and abstract filtering, 52 records were excluded, and 68 were left. Further, 39 articles were excluded from the full-text screening, and 29 articles that met the criteria were included in this review [2, 26–53]. The study flow is depicted in Figure 2.

Participants: a total of 29 studies were included, with a total of 2,666 patients. All trials met the diagnostic criteria; 20 trials mentioned the diagnostic criteria used [2, 27, 32–40, 42–48, 50, 51, 53], and three were confirmed cases from hospitals [26, 28, 29, 31, 41, 49, 52]. None reported polycentric differentiation or syndrome differentiation, as described by traditional Chinese medicine (TCM).

Intervention: this systematic review included 17 interventions (Table 1). Seven of the articles included two experimental groups, including fire needle therapy alone and combined therapy, with one control group each [2, 27, 30, 32, 33, 39, 49].

Comparison: to present the results of the studies more concisely, we developed subgroups based on the different interventions. Seventeen RCTs used fire needle therapy only [2, 26, 27, 29–33, 36, 37, 39, 41, 43, 46–48, 51, 53], whereas ten studies were in combination with TCM [27, 31–33, 35, 37–39, 45, 51]. Four trials used fire needle therapy in combination with immunosuppressive agents [2, 34, 42, 49], one trial used fire needle therapy in combination with imiquimod [28], one used fire needle therapy and tretinoin [44], and the other four used a multidrug combination [29, 40, 48, 52]. The treatment course ranged from 4 to 10 weeks.

Outcome: the primary outcome indicator is efficacy rate. Four trials used the efficacy rate from the skin lesion scores as the outcome indicator [29, 31–33, 43, 53], whereas the other 25 studies used symptom assessment.

Study design: all included studies are RCTs.

### 3.2. Risk of Bias Assessment

**Figure 3:**
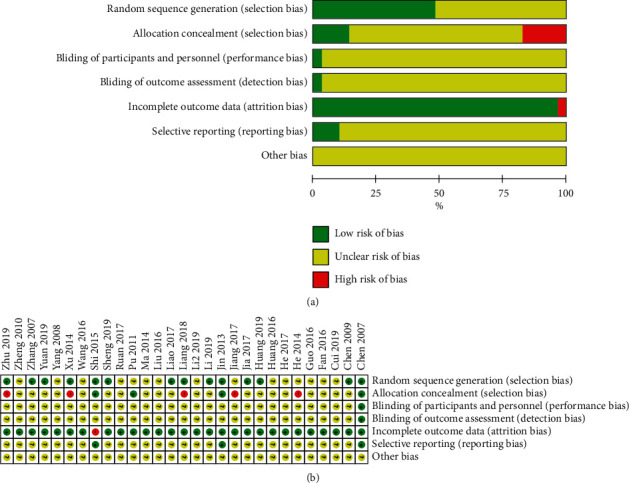
Risk of bias in the included studies for the safety and efficacy of fire needle therapy for flat warts. (a) Risk of bias graph; (b) risk of bias summary.

Fifteen trials reported the generation of random sequences, nine of which used a random number table [2, 26–29, 34, 44, 50, 52], four used a computer random number generator (SAS) [33, 43, 47, 53], and one used a lottery method [31]. One trial used sequential sampling inspection and introduced artificial evaluation to the process of case inclusion, which was then identified as high risk [40]. The other 15 trials mentioned random only, without further explanation; thus, we identified them as unclear. Eight trials used the random concealment method, three of which used sequentially coded opaque envelopes and were thus identified as low-risk [43, 47, 53], whereas five experiments used an open consultation order, which were identified as high risk [28, 33, 36, 44, 46]. Only one study reported the implementation and monitoring of blinding [53]. Since one trial had no explanation regarding the amount of data loss or the reason for data loss, it was considered high risk [43]. Three trials provided detailed protocols and result reports followed by the research plan, and these were identified as low risk [43, 47, 53]. No study described other biases (Figure 3). The risk of publication bias across studies is presented in a funnel plot (Supplementary file 4: Figure S1), implying low-quality methodology and that publication bias related to insufficient sample size may exist.

### 3.3. Level of Evidence

Based on the GRADE system, the evidence on the safety and efficacy of fire needle therapy for flat warts was evaluated using the GRADEpro GDT platform. The evidence of efficacy rate of fire needle alone compared with control groups was level C (Table 2), whereas the evidence of fire needle combined therapies was level B. In addition, the results of secondary indices indicated that the evidence of skin lesion scores and adverse events was level C, and the evidence of cytokine expression levels and recurrence rate was level B. All of them were moderate or weak recommendations.

### 3.4. Primary Outcomes

#### 3.4.1. Efficacy Rate

Twenty-six trials used the clinical evaluation criteria of clinical dermatology that defined the reduction of symptoms or scores of more than 30% as effective, and less than 30% as invalid [25]. One study was based on the evaluation criteria for treatment of flat warts given in the Standards for Diagnosis and Treatment of Traditional Chinese Medical Diseases that defines the degree of skin loss of more than 20% as effective, and otherwise invalid [51]. The evaluation criteria for other two studies were unclear [47, 53]. The results of the meta-analysis showed that the efficacy rate of fire needle therapy alone was higher when compared with that of an immunomodulator (RR = 1.19, 95% CI: 1.03 to 1.37, *I*^2^ = 70%, *P* = 0.02; Table 3; Supplementary file 5: Figure S2) or tretinoin (RR = 1.39, 95% CI: 1.25 to 1.55, *I*^2^ = 0%, *P* < 0.00001). Regarding the different subgroups, the efficacy rate of combination of fire needle therapy with other TCMs was significantly higher than that of TCM alone (RR = 1.16, 95% CI: 1.10 to 1.23, *I*^2^ = 21%, *P* < 0.00001; Supplementary file 6: Figure S3). The groups that used fire needle therapy combined with an immunomodulator (RR = 1.17, 95% CI: 1.07 to 1.28, *I*^2^ = 33%, *P* = 0.0005), imiquimod (RR = 1.21, 95% CI: 1.04 to 1.42, *P* = 0.02), and multidrug therapy (RR = 1.15, 95% CI: 1.07–1.24, *I*^2^ = 0%, *P* = 0.0001) also exhibited statistically significant differences.

When heterogeneity of the subgroups was observed in the efficacy rate of fire needle therapy compared with an immunomodulator, sensitivity analysis was performed (Supplementary file 7: Figure S4). We excluded studies [26] on the sensitivity analysis that led to a reduction in the heterogeneity of the subgroups (*I*^2^ = 0), yet the result was still statistically significant (*P* = 0.006).

### 3.5. Secondary Outcomes

#### 3.5.1. Skin Lesion Scores

Five trials used skin lesion scores as the criteria to assess disease severity [29, 30, 32, 33, 54], as recommended by the Chinese Dermatology Monograph [25]. The overall skin lesion scores of the fire needle group were similar to those of the control group (*P* = 0.15; Table 4; Supplementary file 8: Figure S5), whereas the combined groups had lower scores (SMD = −2.66, 95% CI: −4.55 to −0.78, *I*^*2*^ = 97%, *P* = 0.006; Supplementary file 9: Figure S6). Compared with TCM and tretinoin, the group with fire needle therapy alone had reduced scores with respect to the size (SMD = −1.20, 95% CI: −1.54 to −0.87, *I*^2^ = 0%, *P* < 0.00001), thickness (SMD = −0.94, 95% CI: −1.26 to −0.61, *I*^2^ = 0%, *P* < 0.00001), and itching (SMD = −0.44, 95% CI: −0.75 to −0.13, *I*^2^ = 0%, *P* = 0.006). However, there was no statistically significant difference in the number of warts (*P* = 0.30), color of the skin lesions (*P* = 0.57), and isomorphic response (*P* = 0.23). We conducted a sensitivity analysis of the two subgroups with high heterogeneity, but the result could not be considered significant because there were an insufficient number of trials (Supplementary file 10: Figure S7; Supplementary file 11: Figure S8).

#### 3.5.2. Cytokine Levels

Two studies reported that increased levels of cytokines, including interleukin-2 (IL-2), interleukin-10 (IL-10), and interferon-*γ* (IFN-*γ*), were associated with flat warts [29, 33] (Table 4; Supplementary file 10: Figure S9). After combined treatments, the levels of IL-2 (MD = 5.15, 95% CI: 3.70 to 6.59, *I*^2^ = 0%, *P* < 0.00001) or IFN-*γ* (MD = 7.67, 95% CI: 5.83 to 9.51, *I*^2^ = 0%, *P* < 0.00001) were significantly increased, whereas the level of IL-10 was decreased, compared with that of other drugs (MD = −1.75, 95% CI: −2.45 to −1.05, *I*^2^ = 49%, *P* < 0.00001).

#### 3.5.3. DLQI

One study used DLQI to evaluate the effects of the treatment [33]; it indicated no significant difference when comparing fire needle therapy and TCM (*P* = 0.98; Table 4). However, when compared to the effects of TCM alone, the combination of fire needle therapy and TCM showed a statistical difference (RR = −3.82, 95% CI: −6.32 to −1.32, *P* = 0.003).

#### 3.5.4. Recurrence Rates

Twelve trials reported recurrence rates [2, 28, 29, 31, 34, 40, 43, 49–53]. The follow-up period for 10 studies was 3 months, one was 6 months [49], and one was 12 months [52]. The results of the meta-analysis (Table 4; Supplementary file 13: Figure S10) showed that the recurrence rate in the groups that used fire needle therapy alone was similar to that in the control groups (RR = 0.71, 95% CI: 0.38 to 1.31, *I*^*2*^ = 24%, *P* = 0.27); however, recurrence rates were significantly lower in the combined therapies (RR = 0.34, 95% CI: 0.21 to 0.54, *I*^2^ = 12%, *P* < 0.00001).

#### 3.5.5. Adverse Events

Fourteen trials evaluated the safety of treatment by assessing various types of adverse events [28–31, 35–37, 40, 43, 46, 48–50, 53]. Subgroup analysis (Table 4; Supplementary file 14: Figure S11) showed that patients treated with fire needle therapy were at a reduced risk of erythema (RR = 0.05, 95% CI: 0.00 to 0.90, *P* = 0.04) and blisters (RR = 0.04, 95% CI: 0.00 to 0.71, *P* = 0.03), but were more likely to develop pigmentation (RR = 2.19, 95% CI: 1.15 to 4.17, *I*^2^ = 0%, *P* = 0.02). Furthermore, comparisons of desquamation in the fire needle combined with multidrug therapy group and multidrug therapy alone demonstrated a significant difference (RR = 0.21, 95% CI: 0.07 to 0.64, *I*^2^ = 0%, *P* = 0.006; Supplementary file 15: Figure S12). In the sensitivity analysis, the subgroup of mild burning and pigmentation showed high heterogeneity, which may be related to two studies [30, 50], but the conclusion is still valid with statistical significance (Supplementary file 16: Figure S13; Supplementary file 17: Figure S14).

## 4. Discussion

This systematic review included 29 trials comparing the effectiveness of fire needle therapy alone and combined treatments for flat warts. We found evidence to support that fire needle therapy showed significantly better therapeutic potential than immunomodulators or tretinoin, especially in terms of wart size, thickness, and itching. The reason for this may be related to the mechanism of high temperature directly destroying warts and degenerative proteins and killing viruses in epidermal spinous processes, which are possibly the most common methods of inducing cell death and antigen exposure [8, 40, 55]. Based on the high heterogeneity in the efficacy rate of the subgroup that received fire needle therapy compared with that of the immunomodulator, we found that the intervention frequency might be the determining factor responsible for the differences. The results of the sensitivity analysis resolved the heterogeneity and proved that the results are still significant. However, the efficacy rate between fire needle therapy and liquid nitrogen or photodynamic therapy did not significantly differ, but the evidence was insufficient because each of the comparisons was made using only one study. Furthermore, our results showed that the total efficacy rate of fire needle therapy alone was as effective as TCM, whereas combination therapies significantly improved effectiveness.

In terms of combined therapies, the total efficacy rates of TCM, immunomodulators, imiquimod, or multi drug therapy were significantly improved after using fire needle therapy, with lower skin lesion scores. The mechanism of fire needle combination therapy is still unclear and may be related to thermal effects that improve microcirculation and enhance drug absorption [35, 40]. Additionally, local or systemic cellular immune responses generated by natural killer (NK) cells have been reported as an important mechanism of fire needle therapy for flat warts [2]. In this meta-analysis, we found that groups that received fire needle therapy combined with tretinoin or TCM treatment were more likely to have decreased IL-10 and increased IL-2 or IFN-*γ* levels after peripheral blood testing. Consistent with this result, an experiment confirmed that HPV infection inhibits NK cell activation by fluorescence quantitative polymerase chain reaction (PCR) and western blot analysis of lesion tissues and peripheral blood samples. The peripheral blood of infected patients analyzed using enzyme-linked immunosorbent assays revealed that IL-2 and IFN-*γ* protein levels were significantly lower than those in normal subjects, whereas the IL-10 levels of the patients were higher [54]. The expression levels of IFN-*γ* and IL-2 mRNA were correlated with wart remission, as evidenced by real-time PCR of select punch biopsy specimens, and IL-2 or IFN-*γ* mRNA levels were significantly increased in tissues of effectively treated viral warts [56]. Treatments of flat warts that regulate the levels of these cytokines have been reported, including retinoids [57]. Fire needle therapy may assist retinoids in enhancing this effect, increasing *IL-2* and *IFN-γ* expression and enhancing cell-mediated immune responses. The levels of IL-2 or IFN-*γ* were positively correlated with promoting the activation of NK cells and removing the target cells infected with viruses [58]. As a cell synthesis inhibitor, IL-10 can directly inhibit IFN-*γ* synthesis, thereby reducing their ability to eliminate toxins [59]. The result of the reduction of IL-10 was consistent with the hypothesis of immunosuppression in infected patients [54].

Combined medication significantly reduced the clinical recurrence rate of flat warts. In addition to their destructive effects, the reduction of recurrence rates through fire needle therapy may be related to the regulation of immune function. According to previous reports, the recurrence rate of flat warts after combined therapy was lower than that of fire needle therapy alone, and it is recommended that, after fire needle treatment, antiviral therapy be commenced to further reduce the recurrence rates [29].

Regarding adverse events, we found that patients were less likely to have blisters and erythema when they were treated with fire needle therapy alone, and desquamation was less likely to occur in groups that received combined therapy. The main adverse events caused by the treatment of flat warts were irritating cautery, hypertrophic scar formation, local or systemic infection, itching, allergies, and increased sensitivity to pain [60–62]. Skin lesions, including pigmentation, scar formation, erythema, desquamation, and blistering, were also common [8]. This study included only 10 adverse events. Blisters have been reported as adverse events in cryotherapy, and erythema and desquamation have been reported as adverse events of squaric acid dibutyl ester or diphencyprone therapy [8]. We demonstrated that, to a limited extent, fire needle therapy can be used as an alternative or complementary therapy to avoid these adverse events. Pigmentation was the main adverse reaction to fire needle therapy alone, whereas our results showed that using combined therapies may lower this risk. Furthermore, some preventive measures for pigmentation have been proposed and implemented clinically. For example, the use of asiaticoside ointment could significantly relieve the pigmentation caused by fire needle therapy [63].

The present study is the first systematic review of the efficacy of fire needle treatment for flat warts that follows the PRISMA statement and is formulated according to the PICOS framework. The heterogeneity of each index factor was evaluated using sensitivity analysis to test the stability of the approach. The GRADE system was adopted to evaluate the level of evidence and make the results more credible. Evidence from 29 clinical trials illustrates that using fire needle therapy could significantly improve the efficacy rate with fewer side effects. This article provides evidence and guidance for the clinical practice of fire needles in flat warts.

However, this study had some limitations. First, the quality of trials was not very good; only fourteen trials reported specific randomization, and only nine mentioned allocation concealment. Only one trial reported blinding, and no studies reported other biases. Second, we did not distinguish between flat warts on the hands and faces. Third, flat warts from children and adults were discussed together; the difference among them requires further research.

## 5. Conclusion

In conclusion, fire needle therapy seems to be more effective against flat warts when compared with an immunomodulator or tretinoin and can reduce adverse events, such as blisters and erythema, but may cause pigmentation. Using fire needle therapy in combination with conventional therapies could significantly improve the treatment efficacy rate and lower the risk of desquamation. However, the quality of the included literature was not good, and large multicenter studies with a large number of samples and high-quality RCTs should be conducted to confirm the efficacy and safety of fire needle therapy in the treatment of flat warts.

## Figures and Tables

**Table 1:** Characteristics of the included trials.

Study	Average course duration of disease		Average age (years)		Treatment course	Sample size		Adverse events	
	E	C	E	C		E	C	E	C
Sheng 2019	NR	NR	23.5 (4.5)	23.2 (4.4)	4 w	60	60	NR	NR
Zhu 2019	NR	NR	25.79 (2.52)	25.81 (2.47)	4 w	48	48	Itching (1), mild burning (1)	Infection (2), itching (2), mild burning (3)
Yuan 2019	8.09 (3.11) m	8.25 (3.04) m	25.97 (6.78)	25.36 (7.36)	8 w	39	39	Itching (3), mild burning (5), erythema (2), pigmentation (2)	Itching (4), mild burning (4), erythema (3)
Li 2019	9.0 (3.8) m	8.8 (3.2) m	30.0 (3.9)	31.3 (3.6)	30 d	40	40	Itching (2)	Itching (3)
Li2 2019	NR	NR	26.8 (3.2); 26.4 (3.3)	26.4 (3.1)	4 w	40; 40	40	Scar (1); 0	Scar (2), pigmentation (3)
Huang 2019	NR	NR	NR	NR	10 w	30; 30	30	NR	NR
Cui 2019	21.92 (6.65) m; 22.75 (6.62) m	22.47 (6.74) m	26 (5.87); 24 (6.94)	25 (6.05)	6 w	30; 30	30	NR	NR
Liang 2018	19.11 (6.23) m; 19.43 (5.98) m	18.76 (6.01) m	26.59 (5.38); 25.55 (5.13)	26.30 (4.88)	4 w	30; 30	30	NR	NR
Liao 2017	3.59 (1.91) y;3.35 (1.87) y	3.41 (1.89) y	25 (7); 26 (5)	25 (8)	4 w	30; 30	30	NR	NR	
Jia 2017	NR	NR	NR	NR	4 w	50	50	NR	NR
Ruan 2017	NR	NR	20.5	20.3	4 w	60	60	Pigmentation (6)	0
Jiang 2017	30.4 (6.45) m		24.8 (6.24)		8w	60	55	Pain (6)	0	
He 2017	1.60 y	1.53 y	28.5	27.5	4 w	60	58	Pain (5)	0
Fan 2016	1.60 y	1.53 y	28.5	27.5	4 w	60	58	NR	NR
Wang 2016	NR	NR	20.8; 20.5	20.3	8 w	38; 52	36	NR	NR
Huang 2016	1.8 (1.82) y		26.8 (6.4)		8 w	49	46	0	Mild burning (5), desquamation (5)
Guo 2016	1.8 (0.60) y	1.70 (0.70) y	23.86 (8.92)	22.28 (9.35)	30 d	38	38	NR	NR
Liu 2016	6.9 m	7.2 m	18.3	19.7	4 w	30	30	NR	NR
Shi 2015	30.03 (16.51) m	29.48 (14.18) m	26.46 (6.48)	25.39 (6.27)	30 d	33	32	Infection (2), mild burning (1), pigmentation (4), desquamation (2), isomorphic response (3)	Infection (2), mild burning (1), pigmentation (2), isomorphic response (3)
He 2014	19.48 (12.95) m	20.24 (13.14) m	30.11 (6.47)	29.87 (6.30)	8 w	46	46	NR	NR
Ma 2014	NR	NR	NR	NR	10 d	38	30	NR	NR
Xu 2014	4.8 y	4.5 y	33	31	30 d	30	30	Pain (5)	Pain (3), blister (11)
Jin 2013	32.89 (12.29) m	31.89 (12.91) m	27.06 (9.15)	26.67 (7.89)	4 w	18	18	NR	NR
Pu 2011	18.2 m; 19.6 m	21.5 m	23; 27	26	4 w	32; 29	27	Itching (4), desquamation (9), pigmentation (25); itching (4), desquamation (3), pigmentation (11);	Itching (6), pigmentation (8), desquamation (12),
Zheng 2010	NR	NR	NR	NR	2 w	60; 60	60	0; 0	Itching (2)
Chen 2009	4.8 y	4.5 y	31	31	30 d	72	48	Pigmentation (4), isomorphic response (2)	Mild burning (6), erythema (6)
Yang 2008	2.28 (1.92) y	1.80 (2.57) y	22.75 (7.59)	24.03 (5.69)	4 w	30	30	NR	NR
Zhang 2007	3.5 m	3.55 m	20.3	21.1	30 d	52	57	NR	NR
Chen 2007	30.13 (17.61) m	29.59 (14.28) m	26.56 (6.58)	25.29 (6.37)	30 d	48	48	Infection (2), mild burning (2), pigmentation (4), isomorphic response (2)	Pigmentation (2), desquamation (8), isomorphic response (1), mild burning (8)

Study	FUP (m)	Recurrence		Patients (M/F)		Interventions		Main outcomes
		E	C	E	C	E	C	

Sheng 2019	NR	NR	NR	19/41	25/35	Fire needle	BCG nucleic acid injection	Efficacy rate
Zhu 2019	3	4	13	25/23	26/22	5% Imiquimod + fire needle	5% Imiquimod	Efficacy rate, RER, AEs
Yuan 2019	3	2	3	22/17	25/14	Fire needle + Isotretinoin Erythromycin Gel	Isotretinoin Erythromycin Gel	Efficacy rate + skin lesion scores + IL-2/IL-10/INF-*γ*, RER, AEs
Li 2019	3	NR	NR	13/27	11/29	Fire needle	Tretinoin	Efficacy rate, AEs
Li2 2019	3	4; 0	11	22/18; 23/17	25/15	Fire needle; fire needle + Xiaoyou Decoction	Recombinant human interferon alpha-2b gel	Efficacy rate + skin lesion scores, RER, AEs
Huang 2019	NR	3; 0	8	40/50	Fire needle; Fire needle + Polymyosin injection	Polymyosin injection	Efficacy rate, RER	
Cui 2019	NR	NR	NR	10/20; 13/17	11 /19	Fire needle; Fire needle + Taohongsiwu Decoction	Taohongsiwu Decoction	Efficacy rate
Liang2018	NR	NR	NR	13/17; 14/16	15/15	Fire needle; Fire needle + Xiaoyou Decoction	Xiaoyou Decoction	Efficacy rate + IL-2/IL-10/IFN-*γ*, DLQI
Liao 2017	NR	NR	NR	11/19; 13/17	12/18	Fire needle; fire needle + mild moxibustion	Mild moxibustion	Efficacy rate
Jia 2017	3	7	10	22/28	24/26	Fire needle + recombinant human interferon alpha 2	Recombinant human interferon alpha 2	Efficacy rate, RER
Ruan 2017	NR	NR	NR	20/40	18/42	Fire needle + Chinese medicine inverted film	Chinese medicine inverted film	Efficacy rate
Jiang 2017	NR	NR	NR	52/63	Fire needle	Mannan peptide	Efficacy rate	
He 2017	NR	NR	NR	21/39	22/36	Fire needle + Xiangfu lotion	Xiangfu lotion	Efficacy rate
Fan 2016	NR	NR	NR	21/39	22/36	Fire needle + Xiangfu lotion	Xiangfu lotion	Efficacy rate
Wang 2016	3	NR	NR	15/23; 20/32	14/22	Fire needle; fire needle + Chinese medicine inverted film	Chinese medicine inverted film	Efficacy rate
Huang 2016	3	3	13	NR	NR	Fire needle + Chinese medicine inverted film + Imiquimod	Chinese medicine inverted film + Imiquimod	Efficacy rate, RER, AEs
Guo 2016	NR	NR	NR	22/16	20/18	Fire needle	Recombinant human interferon alpha 2	Efficacy rate
Liu 2016	NR	NR	NR	11/19	15/15	Fire needle + recombinant human interferon alpha 2	Recombinant human interferon alpha 2	Efficacy rate
Shi 2015	3	1	2	18/15	17/15	Fire needle	Tretinoin	Efficacy rate + skin lesion scores, RER, AEs
He 2014	NR	NR	NR	25/21	22/24	Fire needle + Tazarotene gel	Tazarotene gel	Efficacy rate
Ma 2014	NR	NR	NR	23/45		Fire needle + Quyou Decoction	Quyou Decoction	Efficacy rate
Xu 2014	NR	NR	NR	10/20	7/23	Fire needle	Liquid nitrogen freezing	Efficacy rate, AEs
Jin 2013	1.5	NR	NR	9/9	5/13	Fire needle	Photodynamic	Efficacy rate + skin lesion scores + IL-2/IL-10/INF-*γ*
Pu 2011	NR	NR	NR	16/16; 15/14	15/12	Fire needle; fire needle + tretinoin + BCG polysaccharide nucleic acid	Tretinoin + BCG polysaccharide nucleic acid	Efficacy rate, AEs
Zheng 2010	6	10; 0	0	54/126	Fire needle; Fire needle + Utlins injection	Utlins injection	Efficacy rate, RER, AEs	
Chen 2009	3	2	3	33/39	20/28	Fire needle	Tretinoin	Efficacy rate, RER, AEs
Yang 2008	3	1	3	9/21	12/18	Fire needle + decoction	Fire needle + decoction	Efficacy rate, RER
Zhang 2007	12	2	4	19/33	20/37	Fire needle + tretinoin + BCG polysaccharide nucleic acid	Tretinoin + BCG polysaccharide nucleic acid	Efficacy rate, RER
Chen 2007	3	1	2	23/25	20/28	Fire needle	Tretinoin	Efficacy rate + skin lesion scores, RER, AEs

E: experimental group; C: control group; NR: no report; FUP: follow-up period; M: male; F: female; y: years; m: months; w: weeks; d: days; BCG: Bacillus Calmette–Guerin; RER: recurrence rate; AEs: adverse events; DLQI: Dermatology Life Quality Index; IL-2/10: interleukin-2/10; IFN–*γ*: interferon-*γ*. E, experimental group; C, control group; NR, no report; y, years; m, months; w, weeks; d, days.

**Table 2:** Summary of GRADE on the outcomes of the safety and efficacy of fire needle therapy for flat warts.

Certainty assessment					Number of patients				Effect		Certainty	Importance
Number of studies	Study design	Risk of bias	Inconsistency	Indirectness	Imprecision	Other considerations	Fire needle	Control group	Relative (95% CI)	Absolute (95% CI)		
Efficacy rate of fire needle alone vs. control groups												

18	Randomized trials	Serious	Serious	Not serious	Not serious	None	686/749 (91.6%)	424/568 (74.6%)	RR 1.18 (1.09 to 1.28)	134 more per 1,000 (from 67 more to 209 more)	⊕⊕⃝⃝Low	Important

Efficacy rate of fire needle combined therapies vs. control groups												

20	Randomized trials	Serious	Not serious	Not serious	Not serious	None	831/863 (96.3%)	579/694 (83.4%)	RR 1.16 (1.12 to 1.20)	133 more per 1,000 (from 100 more to 167 more)	⊕⊕⊕⃝Moderate	Important

Skin lesions scores												

9	Randomized trials	Serious	Serious	Not serious	Not serious	None	725	619	—	SMD 1 lower (1.43 lower to 0.57 lower)	⊕⊕⃝⃝Low	Important

Cytokine levels												

2	Randomized trials	Serious	Not serious	Not serious	Not serious	None	207	162	—	MD 0.4 higher (0.19 lower to 1 higher)	⊕⊕⊕⃝Moderate	Important

Recurrence rate												

14	Randomized trials	Serious	Not serious	Not serious	Not serious	None	40/621 (6.4%)	72/508 (14.2%)	RR 0.44 (0.30 to 0.63)	79 fewer per 1,000 (from 99 fewer to 52 fewer)	⊕⊕⊕⃝Moderate	Important

Adverse events												

15	Randomized trials	Serious	Serious	Not serious	Not serious	None	123/1794 (6.9%)	109/1478 (7.4%)	RR 0.83 (0.56 to 1.23)	13 fewer per 1,000 (from 32 fewer to 17 more)	⊕⊕⃝⃝Low	Important

CI, confidence interval; RR, risk ratio; SMD, standardized mean difference; MD, mean difference.

**Table 3:** Efficacy rate comparing fire needle and conventional therapies in a quantitative study on the safety and efficacy of fire needle therapy for flat warts.

Trials	Comparisons	Effect estimates (95% CI)		*P* value
1. Fire needle versus control group				
1.1. *Fire needle versus traditional Chinese medicine*				
Wang 2016	Fire needle versus traditional Chinese medicine	RR	1.01 [0.83, 1.23]	
He 2017	Fire needle versus traditional Chinese medicine	RR	1.12 [1.01, 1.24]	
Liao 2017	Fire needle versus traditional Chinese medicine	RR	1.79 [1.02, 3.14]	
Liang 2018	Fire needle versus traditional Chinese medicine	RR	1.00 [0.69, 1.45]	
Cui 2019	Fire needle versus traditional Chinese medicine	RR	1.09 [0.77, 1.55]	
Meta-analysis		RR	1.10 [0.99, 1.21]	0.07

1.2. *Fire needle versus immunomodulator*				
Zheng 2010	Fire needle versus immunomodulator	RR	1.06 [0.92, 1.21]	
Guo 2016	Fire needle versus immunomodulator	RR	1.20 [1.00, 1.44]	
Jiang 2017	Fire needle versus immunomodulator	RR	1.14 [1.00, 1.29]	
Huang 2019	Fire needle versus immunomodulator	RR	1.09 [0.77, 1.55]	
Li 2019	Fire needle versus immunomodulator	RR	1.09 [0.89, 1.33]	
Sheng 2019	Fire needle versus immunomodulator	RR	1.89 [1.40, 2.55]	
Meta-analysis		RR	1.19 [1.03,1.37]	0.02

*1.3 Fire needle versus tretinoin*				
Chen 2007	Fire needle versus tretinoin	RR	1.48 [1.19, 1.84]	
Chen 2009	Fire needle versus tretinoin	RR	1.48 [1.20, 1.84]	
Shi 2015	Fire needle versus tretinoin	RR	1.25 [1.01, 1.56]	
Li 2019	Fire needle versus tretinoin	RR	1.36 [1.09, 1.68]	
Meta-analysis		RR	1.39 [1.25, 1.55]	< 0.00001

1.4. *Fire needle versus liquid nitrogen freezing*				
Xu 2014	Fire needle versus liquid nitrogen freezing	RR	0.96 [0.83, 1.12]	0.64

*1.5 Fire needle versus photodynamic*				
Jin 2013	Fire needle versus photodynamic	RR	1.00 [0.85, 1.17]	1

1.6. *Fire needle versus multi drug therapy*				
Pu 2011	Fire needle versus multidrug therapy	RR	1.20 [0.94, 1.28]	0.15

2. Fire needle combined with conventional therapies versus control group				
*2.1 Fire needle combined with traditional Chinese medicine versus traditional Chinese medicine*				
Yang 2006	Fire needle combined with traditional Chinese medicine versus traditional Chinese medicine	RR	1.29 [0.99, 1.67]	
Ma 2014	Fire needle combined with traditional Chinese medicine versus traditional Chinese medicine	RR	1.33 [1.06, 1.66]	
Fan 2016	Fire needle combined with traditional Chinese medicine versus traditional Chinese medicine	RR	1.12 [1.01, 1.24]	
Wang 2016	Fire needle combined with traditional Chinese medicine versus traditional Chinese medicine	RR	1.18 [0.95, 1.45]	
Ruan 2017	Fire needle combined with traditional Chinese medicine versus traditional Chinese medicine	RR	1.05 [0.98, 1.14]	
He 2017	Fire needle combined with traditional Chinese medicine versus traditional Chinese medicine	RR	1.12 [1.01, 1.24]	
Liao 2017	Fire needle combined with traditional Chinese medicine versus traditional Chinese medicine	RR	1.19 [0.69, 2.05]	
Liang 2018	Fire needle combined with traditional Chinese medicine versus traditional Chinese medicine	RR	1.21 [0.93, 1.57]	
Cui 2019	Fire needle combined with traditional Chinese medicine versus traditional Chinese medicine	RR	1.32 [0.96, 1.80]	
Meta-analysis		RR	1.16 [1.10, 1.23]	< 0.00001

2.2. *Fire needle combined with traditional Chinese medicine versus immunomodulator*				
Li 2019	Fire needle combined with traditional Chinese medicine versus immunomodulator	RR	1.19 [0.98, 1.44]	0.09

*2.3 Fire needle combined with immunomodulator versus immunomodulator*				
Zheng 2010	Fire needle combined with immunomodulator versus immunomodulator	RR	1.08 [0.97, 1.20]	
Liu 2016	Fire needle combined with immunomodulator versus immunomodulator	RR	1.21 [1.00, 1.46]	
Jia 2017	Fire needle combined with immunomodulator versus immunomodulator	RR	1.12 [0.96, 1.31]	
Huang 2019	Fire needle combined with immunomodulator versus immunomodulator	RR	1.50 [1.05, 2.14]	
Meta-analysis		RR	1.17 [1.07, 1.28]	0.0005
2.4. *Fire needle combined with tretinoin versus tretinoin*				
He 2014	Fire needle combined with trunnion versus tretinoin	RR	1.09 [0.99, 1.21]	0.07

2.5. *Fire needle combined with imiquimod versus imiquimod*				
Zhu 2019	Fire needle combined with imiquimod versus imiquimod	RR	1.21 [1.04, 1.42]	0.02

2.6. *Fire needle combined with multidrug therapy versus multidrug therapy*				
Zhang 2007	Fire needle combined with multidrug therapy versus multidrug therapy	RR	1.10 [0.98, 1.23]	
Pu 2011	Fire needle combined with multidrug therapy versus multidrug therapy	RR	1.18 [0.94, 1.49]	
Huang 2016	Fire needle combined with multidrug therapy versus multidrug therapy	RR	1.13 [0.99, 1.30]	
Yuan 2019	Fire needle combined with multidrug therapy versus multidrug therapy	RR	1.25 [1.06, 1.48]	
Meta-analysis		RR	1.15 [1.07, 1.24]	0.0001

**Table 4:** Skin lesion scores, cytokine levels, recurrence rates, and adverse events comparing fire needle and conventional therapies in a quantitative study on the safety and efficacy of fire needle therapy for flat warts.

Trials	Comparisons	Effect Estimates (95% CI)		*P* value
1. Skin lesion scores				

1.1 Fire needle versus control group				

1.1.1 Overall				
Liang 2018	Fire needle versus traditional Chinese medicine	RR	−0.05 [−0.67, 0.57]	
Cui 2019	Fire needle versus traditional Chinese medicine	RR	−4.86 [−5.91, −3.82]	
Li2 2019	Fire needle versus immunomodulator	RR	−0.47 [−1.10, 0.16]	
Meta-analysis		RR	−1.75 [−4.15,0.64]	0.15

1.1.2 Number of warts				
Chen 2007	Fire needle versus tretinoin	RR	−1.31 [−1.75, −0.86]	
Shi 2015	Fire needle versus tretinoin	RR	−0.02 [−0.51, 0.46]	
Meta-analysis		RR	−0.67 [−1.93, 0.59]	0.3

1.1.3 Size				
Chen 2007	Fire needle versus tretinoin	RR	−1.17 [−1.61, −0.74]	
Shi 2015	Fire needle versus tretinoin	RR	−1.25 [−1.79, −0.72]	
Meta-analysis		RR	−1.20 [−1.54, −0.87]	<0.00001

1.1.4 Thickness				
Chen 2007	Fire needle versus tretinoin	RR	−0.97 [−1.39, −0.55]	
Shi 2015	Fire needle versus tretinoin	RR	−0.89 [−1.40, −0.38]	
Meta-analysis		RR	−0.94 [−1.26, −0.61]	< 0.00001

1.1.5 Skin lesion color				
Chen 2007	Fire needle versus tretinoin	RR	−0.89 [−1.31, −0.47]	
Shi 2015	Fire needle versus tretinoin	RR	0.25 [−0.24, 0.74]	
Meta-analysis		RR	−0.32 [−1.44, 0.79]	0.57

1.1.6 Itching				
Chen 2007	Fire needle versus tretinoin	RR	−0.42 [−0.83, −0.02]	
Shi 2015	Fire needle versus tretinoin	RR	−0.47 [−0.96, 0.03]	
Meta-analysis		RR	−0.44 [−0.75, −0.13]	0.006

1.1.7 Isomorphic response				
Chen 2007	Fire needle versus tretinoin	RR	0.17 [−0.23, 0.57]	
Shi 2015	Fire needle versus tretinoin	RR	0.22 [−0.27, 0.71]	
Meta-analysis		RR	0.19 [−0.12, 0.50]	0.23

1.2 Fire needle combined with control group versus control group				

1.2.1 Skin lesions overall scores				
Liang 2018	Fire needle combined with traditional Chinese medicine versus traditional Chinese medicine	RR	−0.54 [−1.18, 0.09]	
Cui 2019	Fire needle combined with traditional Chinese medicine versus traditional Chinese medicine	RR	−0.58 [−1.21, 0.05]	
Li2 2019	Fire needle combined with traditional Chinese medicine versus immunomodulator	RR	−9.84 [−11.74, −7.94]	
Yuan 2019	Fire needle combined with multidrug therapy versus multidrug therapy	RR	−1.09 [−1.57, −0.61]	
Meta-analysis		RR	−2.66 [−4.55, −0.78]	0.006

2. Cytokine levels				

2.1 Fire needle combined with control group versus control group				

2.1.1 Interleukin-2				
Liang 2018	Fire needle combined with traditional Chinese medicine versus traditional Chinese medicine	RR	5.56 [3.05, 8.07]	
Yuan 2019	Fire needle combined with multidrug therapy versus multidrug therapy	RR	4.94 [3.17, 6.71]	
Meta-analysis		RR	5.15 [3.70, 6.59]	<0.00001

2.1.2 Interleukin-10				
Liang 2018	Fire needle combined with traditional Chinese medicine versus traditional Chinese medicine	RR	−2.48 [−3.71, −1.25]	
Yuan 2019	Fire needle combined with multidrug therapy versus multidrug therapy	RR	−1.41 [−2.26, −0.56]	
Meta-analysis		RR	−1.75 [−2.45, −1.05]	<0.00001

2.1.3 Interferon-*γ*				
Liang 2018	Fire needle combined with traditional Chinese medicine versus traditional Chinese medicine	RR	7.75 [4.24, 11.26]	
Yuan 2019	Fire needle combined with multidrug therapy versus multidrug therapy	RR	7.64 [5.48, 9.80]	
Meta-analysis		RR	7.67 [5.83, 9.51]	<0.00001

3 Dermatology Life Quality Index				
3.1 Fire needle versus control group				
Liang 2018	Fire needle versus traditional Chinese medicine	RR	−0.03 [−2.65, 2.59]	0.98

3.1 Fire needle combined with control group versus control group				
Liang 2018	Fire needle combined with traditional Chinese medicine versus traditional Chinese medicine	RR	−3.82 [−6.32, −1.32]	0.003

4. Recurrence rate				

4.1 Fire needle versus control group				
Chen 2007	Fire needle versus tretinoin	RR	0.50 [0.05, 5.33]	
Chen 2009	Fire needle versus tretinoin	RR	0.44 [0.08, 2.56]	
Shi 2015	Fire needle versus tretinoin	RR	0.48 [0.05, 5.09]	
Zheng 2010	Fire needle versus immunomodulator	RR	10.67 [0.65, 176.19]	
Li2 2019	Fire needle versus immunomodulator	RR	0.33 [0.11, 1.05]	
Huang 2019	Fire needle versus immunomodulator	RR	0.38 [0.10, 1.46]	
Meta-analysis		RR	0.71 [0.38, 1.31]	0.27

4.2 Fire needle combined with control group versus control group				
Yang 2008	Fire needle combined with traditional Chinese medicine versus traditional Chinese medicine	RR	0.33 [0.04, 3.03]	
Li2 2019	Fire needle combined with traditional Chinese medicine versus immunomodulator	RR	0.05 [0.00, 0.80]	
Huang 2016	Fire needle combined with immunomodulator versus immunomodulator	RR	0.22 [0.07, 0.71]	
Huang 2019	Fire needle combined with immunomodulator versus immunomodulator	RR	0.06 [0.00, 1.00]	
Zhu 2019	Fire needle combined with imiquimod versus imiquimod	RR	0.31 [0.11, 0.88]	
Zhang 2007	Fire needle combined with multidrug therapy versus multidrug therapy	RR	0.55 [0.10, 2.87]	
Jia 2017	Fire needle combined with multidrug therapy versus multidrug therapy	RR	0.70 [0.29, 1.69]	
Yuan 2019	Fire needle combined with multidrug therapy versus multidrug therapy	RR	0.84 [0.15, 4.77]	
Meta-analysis		RR	0.34 [0.21, 0.54]	<0.00001

5. Adverse events				

5.1 Fire needle versus control group				

5.1.1 Infection				
Chen 2007	Fire needle versus tretinoin	RR	5.00 [0.25, 101.48]	
Shi 2015	Fire needle versus tretinoin	RR	0.97 [0.15, 6.47]	
meta-analysis		RR	1.55 [0.31, 7.71]	0.59

5.1.2 Itching				
Zheng 2010	Fire needle versus immunomodulator	RR	0.10 [0.00, 2.07]	
Pu 2011	Fire needle versus multidrug therapy	RR	0.54 [0.14, 2.09]	
Li 2019	Fire needle versus tretinoin	RR	0.67 [0.12, 3.78]	
Meta-analysis		RR	0.48 [0.18, 1.32]	0.15

5.1.3 Pain				
Xu 2014	Fire needle versus liquid nitrogen freezing	RR	1.67 [0.44, 6.36]	
He 2017	Fire needle versus traditional Chinese medicine	RR	1−.64 [0.60, 188.18]	
Jiang 2017	Fire needle versus immunomodulator	RR	11.93 [0.69, 207.04]	
Meta-analysis		RR	3.73 [0.87, 16.06]	0.08

5.1.4 Mild burning				
Chen 2007	Fire needle versus tretinoin	RR	5.00 [0.25, 101.48]	
Chen 2009	Fire needle versus tretinoin	RR	0.05 [0.00, 0.90]	
Shi 2015	Fire needle versus tretinoin	RR	0.97 [0.06, 14.85]	
Meta-analysis		RR	0.61 [0.04, 8.47]	0.72

5.1.5 Erythema				
Chen 2009	Fire needle versus tretinoin	RR	0.05 [0.00, 0.90]	0.04

5.1.6 Pigmentation				
Chen 2007	Fire needle versus tretinoin	RR	2.00 [0.38, 10.41]	
Chen 2009	Fire needle versus tretinoin	RR	6.04 [0.33, 109.71]	
Shi 2015	Fire needle versus tretinoin	RR	1.94 [0.38, 9.86]	
Pu 2011	Fire needle versus multidrug therapy	RR	2.54 [1.10, 5.86]	
Li 2019	Fire needle versus immunomodulator	RR	0.17 [0.01, 4.01]	
Meta-analysis		RR	2.19 [1.15, 4.17]	0.02

5.2 Fire needle combined with control group versus control group				
5.2.1 Infection				
Zhu 2019	Fire needle combined with imiquimod versus imiquimod	RR	0.20 [0.01, 4.06]	0.29

5.2.2 Itching				
Zhu 2019	Fire needle combined with imiquimod versus imiquimod	RR	0.50 [0.05, 5.33]	
Pu 2011	Fire needle combined with multidrug therapy versus multidrug therapy	RR	0.64 [0.17, 2.49]	
Yuan 2019	Fire needle combined with multidrug therapy versus multidrug therapy	RR	0.75 [0.18, 3.13]	
Meta-analysis		RR	0.66 [0.27, 1.64]	0.37

5.2.3 Mild burning				
Zhu 2019	Fire needle combined with imiquimod versus imiquimod	RR	0.33 [0.04, 3.09]	
Huang 2016	Fire needle combined with multidrug therapy versus multidrug therapy	RR	0.09 [0.00, 1.50]	
Yuan 2019	Fire needle combined with multidrug therapy versus multidrug therapy	RR	1.25 [0.36, 4.31]	
Meta-analysis		RR	0.48 [0.10, 2.31]	0.36

5.2.4 Erythema				
Yuan 2019	Fire needle combined with multidrug therapy versus multidrug therapy	RR	0.67 [0.12, 3.77]	0.65

5.2.5 Pigmentation				
Ruan 2017	Fire needle combined with traditional Chinese medicine versus traditional Chinese medicine	RR	13.00 [0.75, 225.75]	
Li2 2019	Fire needle combined with traditional Chinese medicine versus immunomodulator	RR	0.10 [0.01, 2.04]	
Pu 2011	Fire needle combined with multidrug therapy versus multidrug therapy	RR	1.33 [0.51, 3.43]	
Yuan 2019	Fire needle combined with multi drug therapy versus multidrug therapy	RR	5.00 [0.25, 100.89]	
Meta-analysis		RR	1.65 [0.32, 8.48]	0.55

5.2.6 Desquamation				
Pu 2011	Fire needle combined with multidrug therapy versus multidrug therapy	RR	0.24 [0.07, 0.83]	
Huang 2016	Fire needle combined with multidrug therapy versus multidrug therapy	RR	0.09 [0.00, 1.50]	
Meta-analysis		RR	0.21 [0.07, 0.64]	0.006

## Data Availability

All of the data used to support the findings of this study are available from the corresponding author upon request.

## Abbreviations

CD4: Cluster of differentiation 4
CI: Confidence intervals
CNKI: China National Knowledge Infrastructure
SinoMed: Chinese Biomedical Database
VIP: China Science and Technology Journal Database
DLQI: Dermatology Life Quality Index
HPV: Human papillomavirus
IL-2: Interleukin-2
IL-10: Interleukin-10
IFN-*γ*: interferon-*γ*
MD: Mean difference
NK: Natural killer
PCR: Polymerase chain reaction
PRISMA: Preferred Reporting Items for Systematic Reviews and Meta-Analyses
RCT: Randomized controlled trial
GRADE: Grades according to grades of recommendation, assessment, development, and evaluation working group
GDT: Guideline development tool
RR: Risk ratios
SMD: Standard mean difference
TCM: Traditional Chinese medicine
PICOS: Participants, intervention, comparison, outcome, and study design.

## Supplementary Materials

Supplementary Materials Supplementary Table S1: PRISMA checklist. PRISMA: Preferred Reporting Items for Systematic Reviews and Meta-Analyses. Supplementary Table S2: search strategy of electronic databases in sequence. Supplementary Table S3: participants, intervention, comparison, outcome, and study design criteria (PICOS) for inclusion and exclusion of studies. Supplementary Figure S1: funnel plot of a quantitative study on the safety and efficacy of fire needle therapy for flat warts. Supplementary Figure S2: forest plot of the efficacy rates comparing fire needle therapy alone and control groups in a quantitative study on the efficacy of fire needle therapy for flat warts. Supplementary Figure S3: forest plot of the efficacy rate comparing fire needle combined therapies and control groups in a quantitative study on the efficacy of fire needle therapy for flat warts. Supplementary Figure S4: forest plot of the efficacy rates comparing fire needle therapy alone and control groups in a quantitative study on the efficacy of fire needle therapy for flat warts (sensitivity analysis). Supplementary Figure S5: forest plot comparing symptom scores between fire needle therapy alone and control groups in a quantitative study on the efficacy of fire needle therapy for flat warts. Supplementary Figure S6: forest plot comparing overall symptom scores between fire needle combined therapies and control groups in a quantitative study on the efficacy of fire needle therapy for flat warts. Supplementary Figure S7: forest plot comparing symptom scores between fire needle therapy alone and control groups in a quantitative study on the efficacy of fire needle therapy for flat warts (sensitivity analysis). Supplementary Figure S8: forest plot comparing overall symptom scores between fire needle combined therapies and control groups in a quantitative study on the efficacy of fire needle therapy for flat warts (sensitivity analysis). Supplementary Figure S9: forest plot comparing cytokine expression levels between fire needle therapy combined with TCM or tretinoin and control groups in a quantitative study on the efficacy of fire needle therapy for flat warts. Supplementary Figure S10: forest plot comparing recurrence rates between fire needle therapy alone and control groups in a quantitative study on the efficacy of fire needle therapy for flat warts. Supplementary Figure S11: forest plot comparing adverse events between fire needle therapy alone and control groups in a quantitative study on the safety of fire needle therapy for flat warts. Supplementary Figure S12: forest plot comparing adverse events between fire needle combined therapies and control groups in a quantitative study on the safety of fire needle therapy for flat warts. Supplementary Figure S13: forest plot comparing adverse events between fire needle therapy alone and control groups in a quantitative study on the safety of fire needle therapy for flat warts (sensitivity analysis). Supplementary Figure S14: forest plot comparing adverse events between fire needle combined therapies and control groups in a quantitative study on the safety of fire needle therapy for flat warts (sensitivity analysis).
