# Supplementary material for: Efficacy and Safety of Fire Needle Therapy for Flat Warts: Evidence from 29 Randomized Controlled Trials
Source: Evid Based Complement Alternat Med. 2021 Jan 16;2021:9513762. doi: 10.1155/2021/9513762 (PMC7834788; doi:10.1155/2021/9513762)
Supplement: Supplementary Materials — Supplementary Table S1: PRISMA checklist. PRISMA: Preferred Reporting Items for Systematic Reviews and Meta-Analyses. Supplementary Table S2: search strategy of electronic databases in sequence. Supplementary Table S3: participants, intervention, comparison, outcome, and study design criteria (PICOS) for inclusion and exclusion of studies. Supplementary Figure S1: funnel plot of a quantitative study on the safety and efficacy of fire needle therapy for flat warts. Supplementary Figure S2: forest plot of the efficacy rates comparing fire needle therapy alone and control groups in a quantitative study on the efficacy of fire needle therapy for flat warts. Supplementary Figure S3: forest plot of the efficacy rate comparing fire needle combined therapies and control groups in a quantitative study on the efficacy of fire needle therapy for flat warts. Supplementary Figure S4: forest plot of the efficacy rates comparing fire needle therapy alone and control groups in a quantitative study on the efficacy of fire needle therapy for flat warts (sensitivity analysis). Supplementary Figure S5: forest plot comparing symptom scores between fire needle therapy alone and control groups in a quantitative study on the efficacy of fire needle therapy for flat warts. Supplementary Figure S6: forest plot comparing overall symptom scores between fire needle combined therapies and control groups in a quantitative study on the efficacy of fire needle therapy for flat warts. Supplementary Figure S7: forest plot comparing symptom scores between fire needle therapy alone and control groups in a quantitative study on the efficacy of fire needle therapy for flat warts (sensitivity analysis). Supplementary Figure S8: forest plot comparing overall symptom scores between fire needle combined therapies and control groups in a quantitative study on the efficacy of fire needle therapy for flat warts (sensitivity analysis). Supplementary Figure S9: forest plot comparing cytokine expression levels bet [file 9513762.f1.doc]

# Supplementary Information

**Efficacy and Safety of Fire Needle Therapy for Flat Warts: Evidence from 29 Randomized Controlled Trials**

Ying Zhang^1†^, Jing-si Jiang^1†^, Le Kuai^1^, Yue Luo^1^, Jia-le Chen^1^, Yan-jiao Wang^1^, Rong Xu^1^, Meng Xing^1^, Liu Liu^1^, Xin Li^1,2*^, and Bin Li^1, 2, 3*^

Supplementary Table S1: PRISMA checklist

| **Section/topic** | **#** | **Checklist item** | **Reported on page #** |
| --- | --- | --- | --- |
| **TITLE** | | |  |
| Title | 1 | Identify the report as a systematic review, meta-analysis, or both. | **Page 1 (Title)** |
| **ABSTRACT** | | |  |
| Structured summary | 2 | Provide a structured summary including, as applicable: background; objectives; data sources; study eligibility criteria, participants, and interventions; study appraisal and synthesis methods; results; limitations; conclusions and implications of key findings; systematic review registration number. | **Pages 2 (Abstract)** |
| **INTRODUCTION** | | |  |
| Rationale | 3 | Describe the rationale for the review in the context of what is already known. | **Page 3 (Introduction)** |
| Objectives | 4 | Provide an explicit statement of questions being addressed with reference to participants, interventions, comparisons, outcomes, and study design (PICOS). | **Page 4 (Introduction)** |
| **METHODS** | | |  |
| Protocol and registration | 5 | Indicate if a review protocol exists, if and where it can be accessed (e.g., Web address), and, if available, provide registration information including registration number. | **Page 5 (Registration)** |
| Eligibility criteria | 6 | Specify study characteristics (e.g., PICOS, length of follow-up) and report characteristics (e.g., years considered, language, publication status) used as criteria for eligibility, giving rationale. | **Page 5 (Search trails)** |
| Information sources | 7 | Describe all information sources (e.g., databases with dates of coverage, contact with study authors to identify additional studies) in the search and date last searched. | **Page 5 (Search trails)** |
| Search | 8 | Present full electronic search strategy for at least one database, including any limits used, such that it could be repeated. | **Page 5 (Search trails)** |
| Study selection | 9 | State the process for selecting studies (i.e., screening, eligibility, included in systematic review, and, if applicable, included in the meta-analysis). | **Page 6 (Study selection)** |
| Data collection process | 10 | Describe method of data extraction from reports (e.g., piloted forms, independently, in duplicate) and any processes for obtaining and confirming data from investigators. | **Page 6 (Data extraction)** |
| Data items | 11 | List and define all variables for which data were sought (e.g., PICOS, funding sources) and any assumptions and simplifications made. | **Page 6 (Data extraction)** |
| Risk of bias in individual studies | 12 | Describe methods used for assessing risk of bias of individual studies (including specification of whether this was done at the study or outcome level), and how this information is to be used in any data synthesis. | **Page 6 (****Risk of bias assessment)** |
| Summary measures | 13 | State the principal summary measures (e.g., risk ratio, difference in means). | **Page 7 (****Statistical analysis)** |
| Synthesis of results | 14 | Describe the methods of handling data and combining results of studies, if done, including measures of consistency (e.g., I^2^) for each meta-analysis. | **Page 7 (Statistical analysis)** |

| **Section/topic** | **#** | **Checklist item** | **Reported on page #** |
| --- | --- | --- | --- |
| Risk of bias across studies | 15 | Specify any assessment of risk of bias that may affect the cumulative evidence (e.g., publication bias, selective reporting within studies). | **Page 6 (Risk of bias assessment)** |
| Additional analyses | 16 | Describe methods of additional analyses (e.g., sensitivity or subgroup analyses, meta-regression), if done, indicating which were pre-specified. | **Page 7 (****Statistical analysis)** |
| **RESULTS** | | |  |
| Study selection | 17 | Give numbers of studies screened, assessed for eligibility, and included in the review, with reasons for exclusions at each stage, ideally with a flow diagram. | **Page 8 (Included studies and the characteristics; Figure 1)** |
| Study characteristics | 18 | For each study, present characteristics for which data were extracted (e.g., study size, PICOS, follow-up period) and provide the citations. | **Page 8 (Table 1; Included studies and the characteristics)** |
| Risk of bias within studies | 19 | Present data on risk of bias of each study and, if available, any outcome level assessment (see item 12). | **Pages 8-9 (Risk of bias assessment, Figure 2)** |
| Results of individual studies | 20 | For all outcomes considered (benefits or harms), present, for each study: (a) simple summary data for each intervention group (b) effect estimates and confidence intervals, ideally with a forest plot. | **Pages 9-11 (Table 2; Table 3; Primary outcomes / Secondary outcomes)** |
| Synthesis of results | 21 | Present results of each meta-analysis done, including confidence intervals and measures of consistency. | **Pages 9-11 (****Primary outcomes / Secondary outcomes)** |
| Risk of bias across studies | 22 | Present results of any assessment of risk of bias across studies (see Item 15). | **Pages 8-9 (Risk of bias assessment)** |
| Additional analysis | 23 | Give results of additional analyses, if done (e.g., sensitivity or subgroup analyses, meta-regression [see Item 16]). | **Pages 9-10 (Primary outcomes)** |
| **DISCUSSION** | | |  |
| Summary of evidence | 24 | Summarize the main findings including the strength of evidence for each main outcome; consider their relevance to key groups (e.g., healthcare providers, users, and policy makers). | **Page 12 (Discussion)** |
| Limitations | 25 | Discuss limitations at study and outcome level (e.g., risk of bias), and at review-level (e.g., incomplete retrieval of identified research, reporting bias). | **Page 14 (Discussion)** |
| Conclusions | 26 | Provide a general interpretation of the results in the context of other evidence, and implications for future research. | **Page 15 (Conclusion)** |
| **FUNDING** | | |  |
| Funding | 27 | Describe sources of funding for the systematic review and other support (e.g., supply of data); role of funders for the systematic review. | **Page 16 (Funding)** |

PRISMA: Preferred Reporting Items for Systematic Reviews and Meta-Analyses

Supplementary Table S2: Search strategy of electronic databases in sequence

| Database | Search strategy of electronic database in sequence | |
| --- | --- | --- |
| Pubmed | 1. | Fire needle [Mesh] |
|  | 2. | Fire needle [Title/Abstract] OR fire needling [Title/Abstract] OR heat needle [Title/Abstract] OR milli-fire needle [Title/Abstract] OR ignipuncture [Title/Abstract] OR pyropuncture [Title/Abstract] OR caloripuncture [Title/Abstract] OR pyronyxis [Title/Abstract] |
|  | 3. | 1 or 2 |
|  | 4. | Flat wart [Mesh] |
|  | 5. | Flat wart [Title/Abstract] OR verruca plana [Title/Abstract] OR plane wart [Title/Abstract] OR wart [Title/Abstract] |
|  | 6. | 4 or 5 |
|  | 7. | 3 and 6 |
| Embase | 1. | Fire needle *'*/exp |
|  | 2. | Fire needle*'* OR fire needling*'* OR heat needle*'* OR milli-fire needle*'* OR ignipuncture*'* OR pyropuncture*'* OR caloripuncture*'* OR pyronyxis*'* |
|  | 3. | 1 or 2 |
|  | 4. | Flat wart*'*/exp |
|  | 5. | Flat wart*'* OR verruca plana*'* OR plane wart*'* OR wart*'* |
|  | 6. | 4 or 5 |
|  | 7. | 3 and 6 |
| Cochrane Central Register of Controlled Trials | 1. | MeSH descriptor: [Fire needle] explode all trees |
|  | 2. | Fire needle:ti,ab,kw OR fire needling:ti,ab,kw OR heat needle:ti,ab,kw OR milli-fire needle:ti,ab,kw OR ignipuncture:ti,ab,kw OR pyropuncture:ti,ab,kw OR caloripuncture:ti,ab,kw OR pyronyxis:ti,ab,kw |
|  | 3. | 1 or 2 |
|  | 4. | MeSH descriptor: [Flat wart] explode all trees |
|  | 5. | Flat wart:ti,ab,kw OR verruca plana:ti,ab,kw OR plane wart:ti,ab,kw OR wart:ti,ab,kw |
|  | 6. | 4 or 5 |
|  | 7. | 3 and 6 |
| CNKI & CQVIP & Wanfang & CBM | 1. | 火针:ti,ab,kw |
|  | 2. | 毫火针:ti,ab,kw |
|  | 3. | 1 or 2 |
|  | 4. | 扁平疣:ti,ab,kw |
|  | 5. | 3 and 4 |

Supplementary Table S3: Participants, intervention, comparison, outcome, and study design criteria (PICOS) for inclusion and exclusion of studies

| **Items** | **Descriptions** |
| --- | --- |
| Participants | Patients diagnosed with flat warts |
| Intervention | Fire needle alone or combined therapies |
| Comparison | Control groups of conventional therapies |
| Outcomes | Standardized therapeutic evaluation (efficacy rate) as the outcome |
| Study design | Randomized controlled trials |

Supplementary Figure S1: Funnel plot graph in a quantitative study on the safety and efficacy of fire needle therapy for flat warts


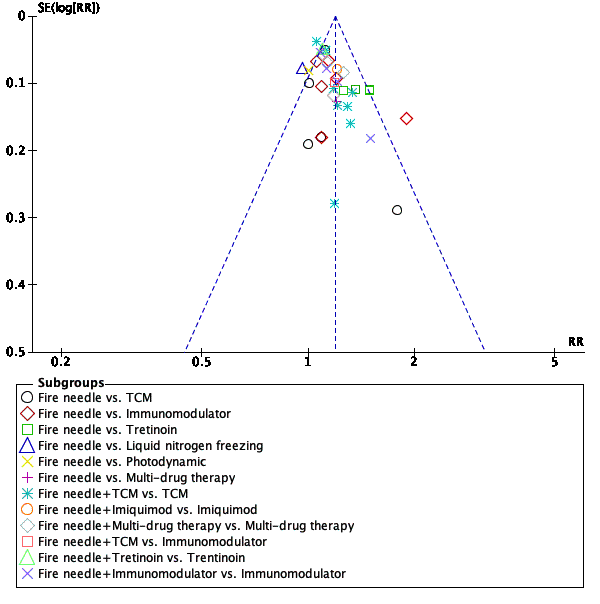


TCM: traditional Chinese medicine.

Supplementary Figure S2: Forest plot of the efficacy rates comparing fire needle therapy alone and control groups in a quantitative study on the efficacy of fire needle therapy for flat warts

**
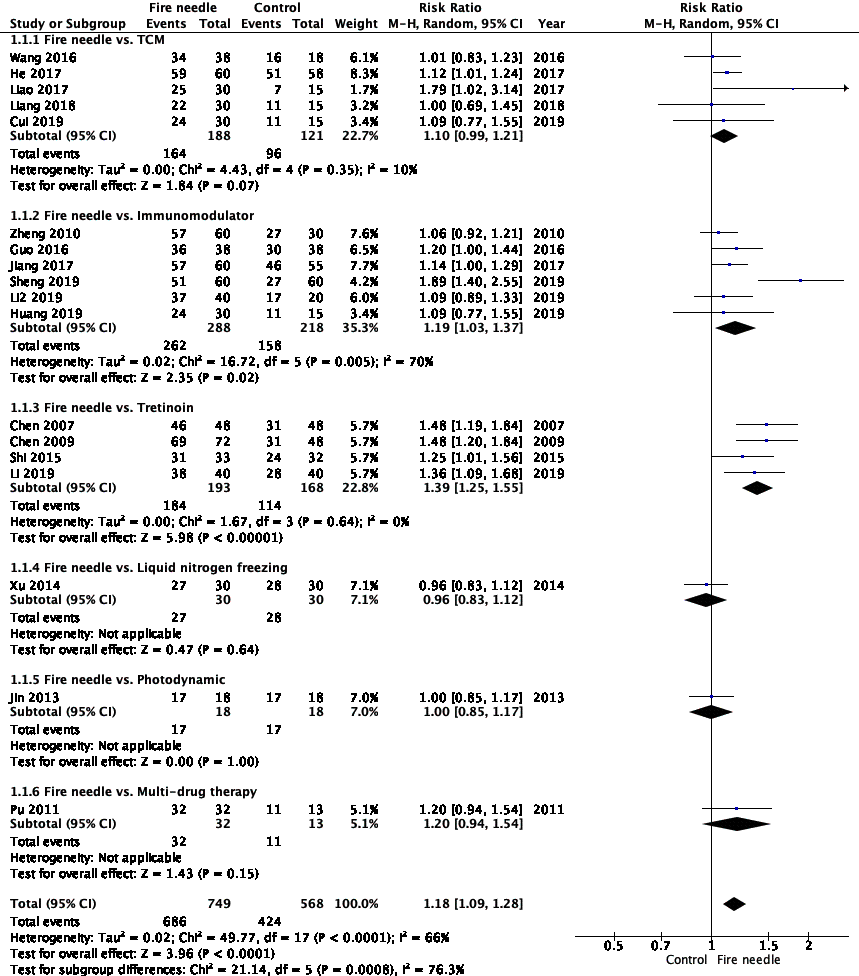
**

TCM: traditional Chinese medicine; CI: indicates confidence interval.

Supplementary Figure S3: Forest plot of the efficacy rate comparing fire needle combined therapies and control groups in a quantitative study on the efficacy of fire needle therapy for flat warts


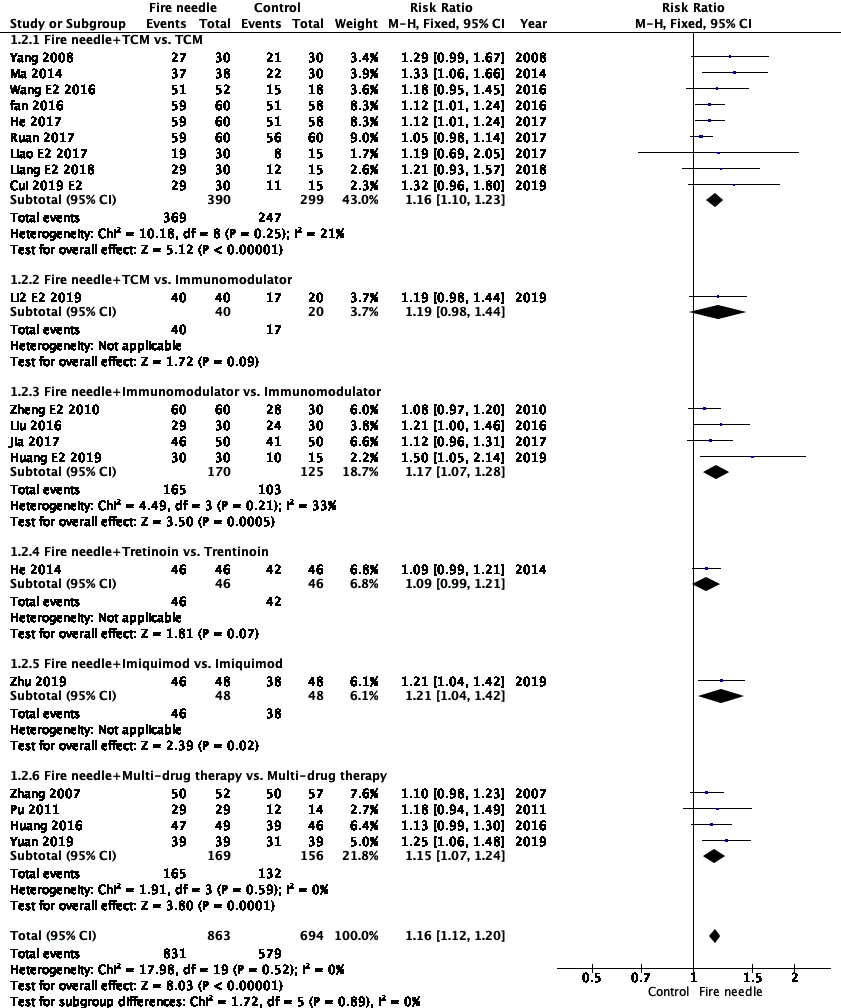


TCM: traditional Chinese medicine; CI: indicates confidence interval.

Supplementary Figure S4: Forest plot of the efficacy rates comparing fire needle therapy alone and control groups in a quantitative study on the efficacy of fire needle therapy for flat warts (sensitivity analysis)


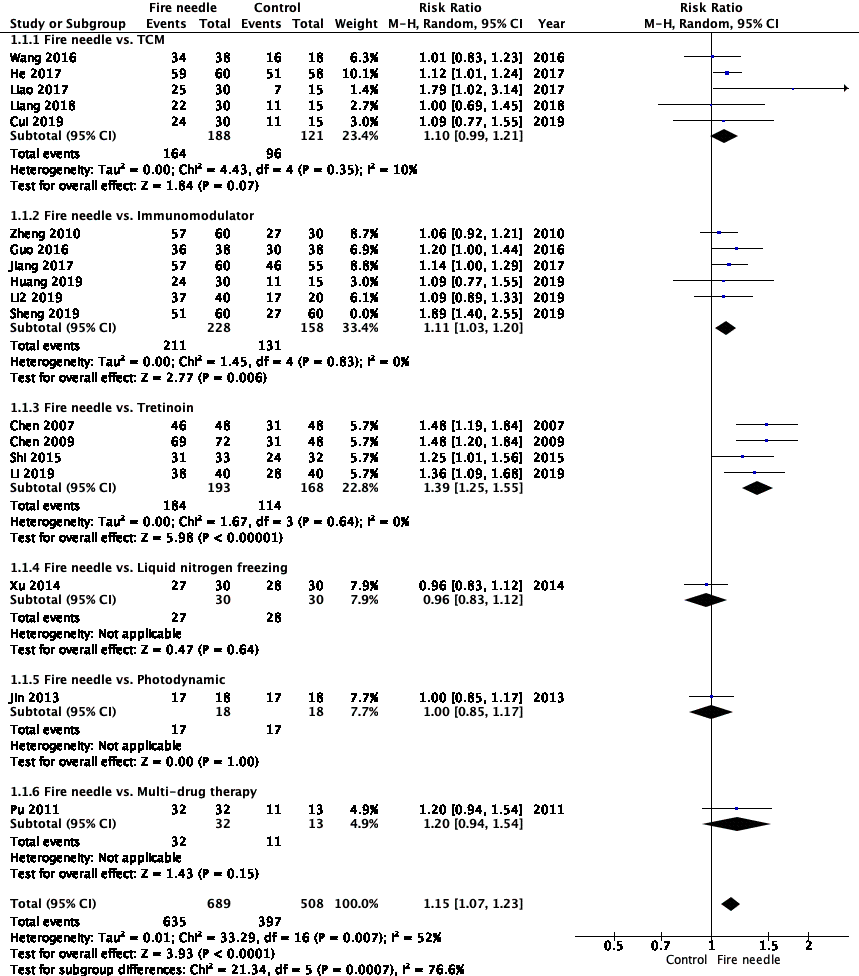


TCM: traditional Chinese medicine; CI: indicates confidence interval

Supplementary Figure S5: Forest plot comparing symptom scores between fire needle therapy alone and control groups in a quantitative study on the efficacy of fire needle therapy for flat warts


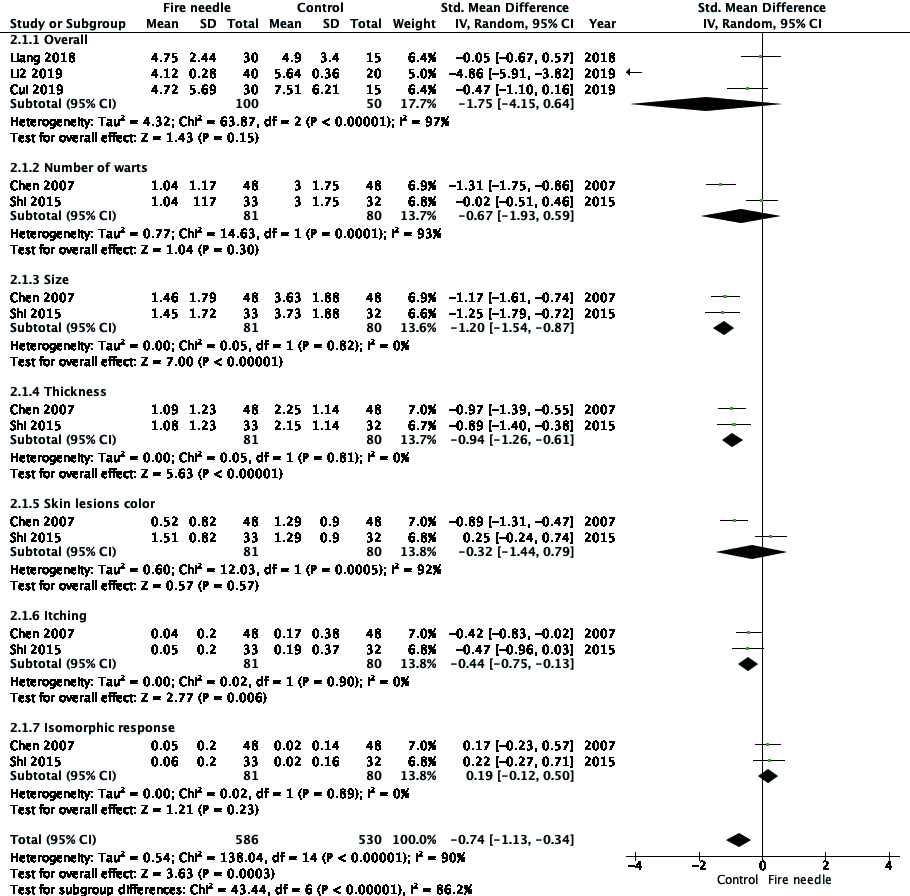


Control groups: TCM and tretinoin. TCM: traditional Chinese medicine; CI: indicates confidence interval.

Supplementary Figure S6: Forest plot comparing overall symptom scores between fire needle combined therapies and control groups in a quantitative study on the efficacy of fire needle therapy for flat warts


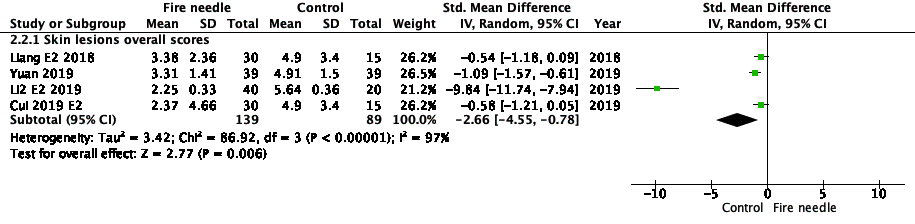


Control groups: TCM and multi-drug therapy. TCM: traditional Chinese medicine; CI: indicates confidence interval

Supplementary Figure S7: Forest plot comparing symptom scores between fire needle therapy alone and control groups in a quantitative study on the efficacy of fire needle therapy for flat warts (sensitivity analysis)


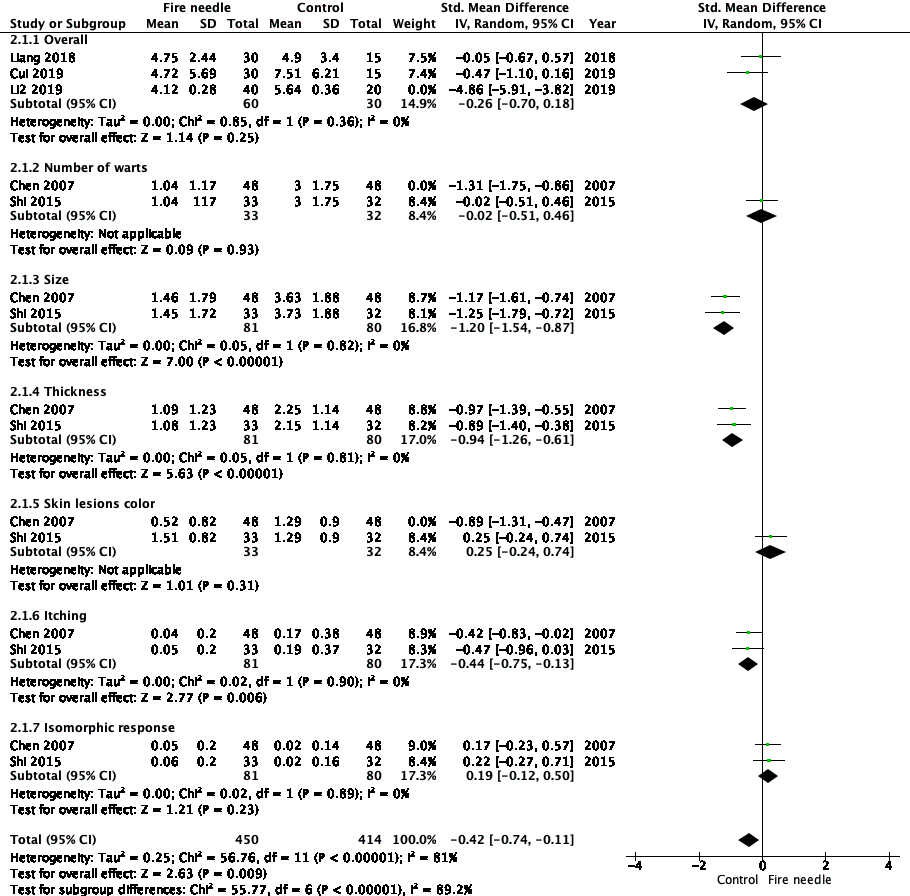


Control groups: TCM and tretinoin. TCM: traditional Chinese medicine; CI: indicates confidence interval.

Supplementary Figure S8: Forest plot comparing overall symptom scores between fire needle combined therapies and control groups in a quantitative study on the efficacy of fire needle therapy for flat warts (sensitivity analysis)


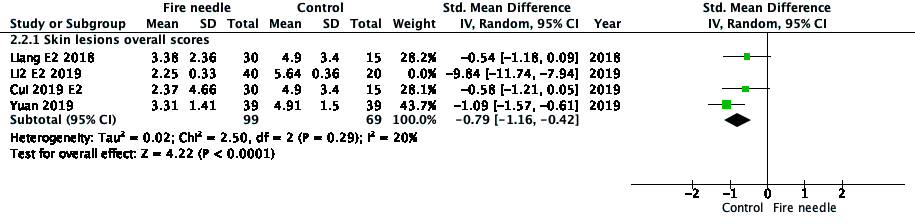


Control groups: TCM and multi-drug therapy. TCM: traditional Chinese medicine; CI: indicates confidence interval

Supplementary Figure S9: Forest plot comparing cytokine expression levels between fire needle therapy combined with TCM or tretinoin and control groups in a quantitative study on the efficacy of fire needle therapy for flat warts


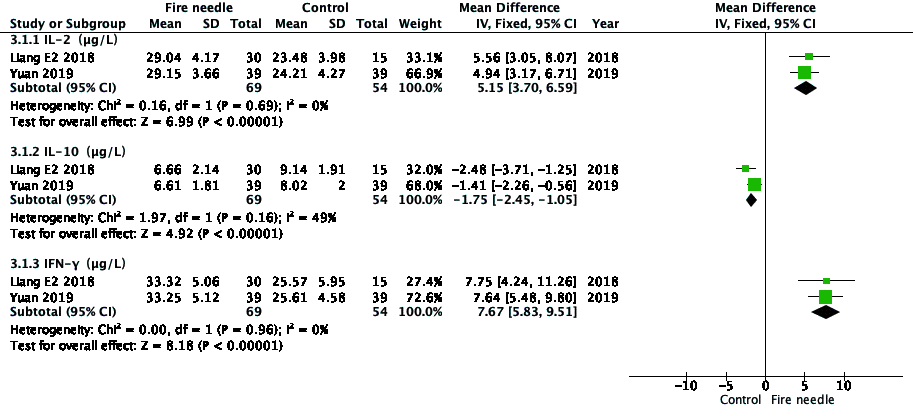


Control groups: TCM or tretinoin. TCM: traditional Chinese medicine; CI: indicates confidence interval. IL-2/10: interleukin-2/10; IFN-γ: interferon-γ

Supplementary Figure S10: Forest plot comparing recurrence rates between fire needle therapy alone and control groups in a quantitative study on the efficacy of fire needle therapy for flat warts


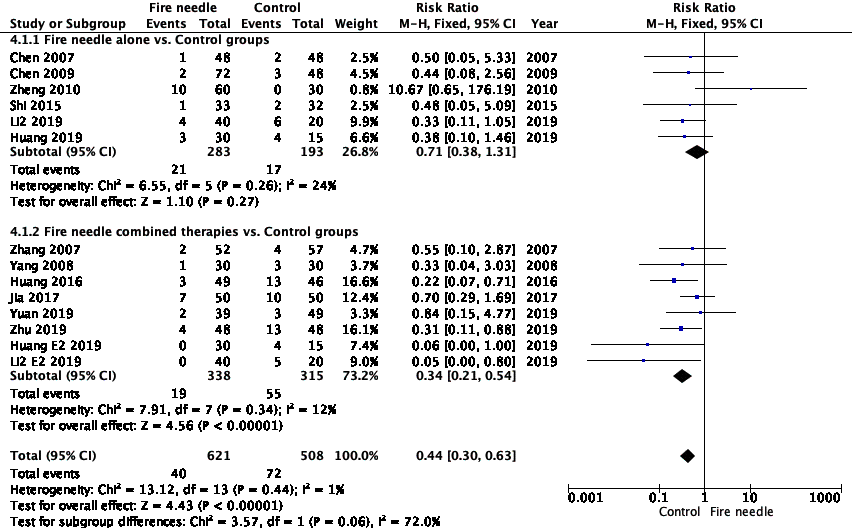


Control groups of 4.1.1: tretinoin and immunomodulator; Control groups of 4.1.2: multi-drug therapy, TCM, immunomodulator and imiquimod. TCM: traditional Chinese medicine; CI: indicates confidence interval.

Supplementary Figure S11: Forest plot comparing adverse events between fire needle therapy alone and control groups in a quantitative study on the safety of fire needle therapy for flat wart


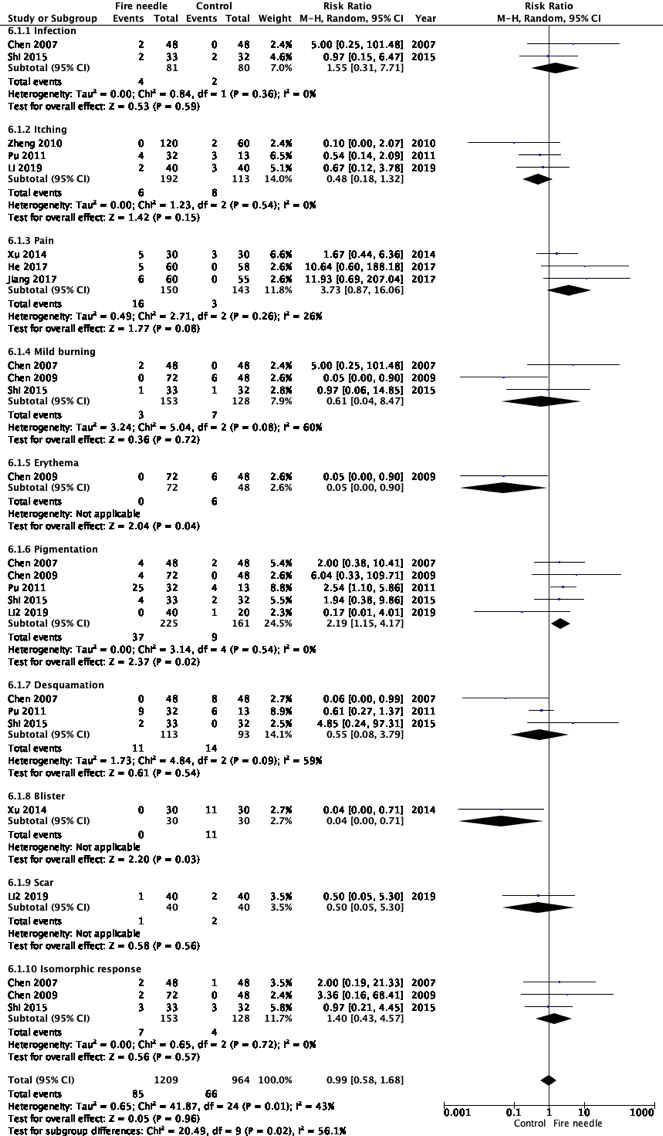


Control groups: tretinoin, immunomodulator, multi-drug therapy, liquid nitrogen freezing, and TCM. TCM: traditional Chinese medicine; CI: indicates confidence interval.

Supplementary Figure S12: Forest plot comparing adverse events between fire needle combined therapies and control groups in a quantitative study on the safety of fire needle therapy for flat warts


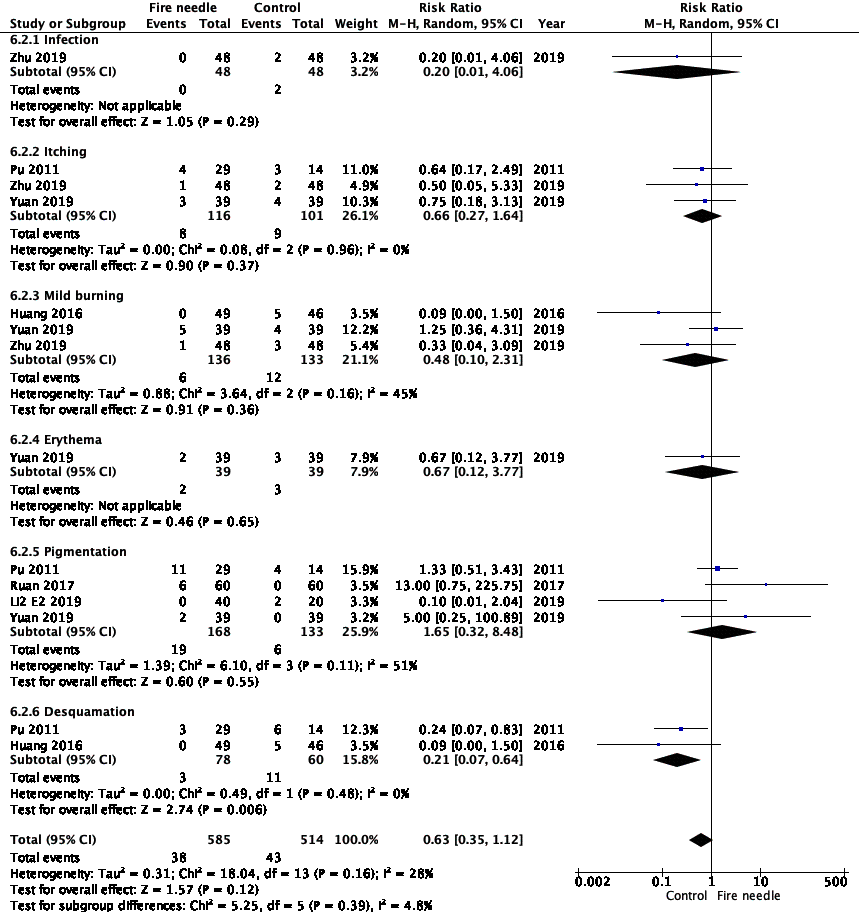


Control groups: imiquimod, multi-drug therapy, TCM, and immunomodulator. TCM: traditional Chinese medicine; CI: indicates confidence interval.

Supplementary Figure S13: Forest plot comparing adverse events between fire needle therapy alone and control groups in a quantitative study on the safety of fire needle therapy for flat warts (sensitivity analysis)


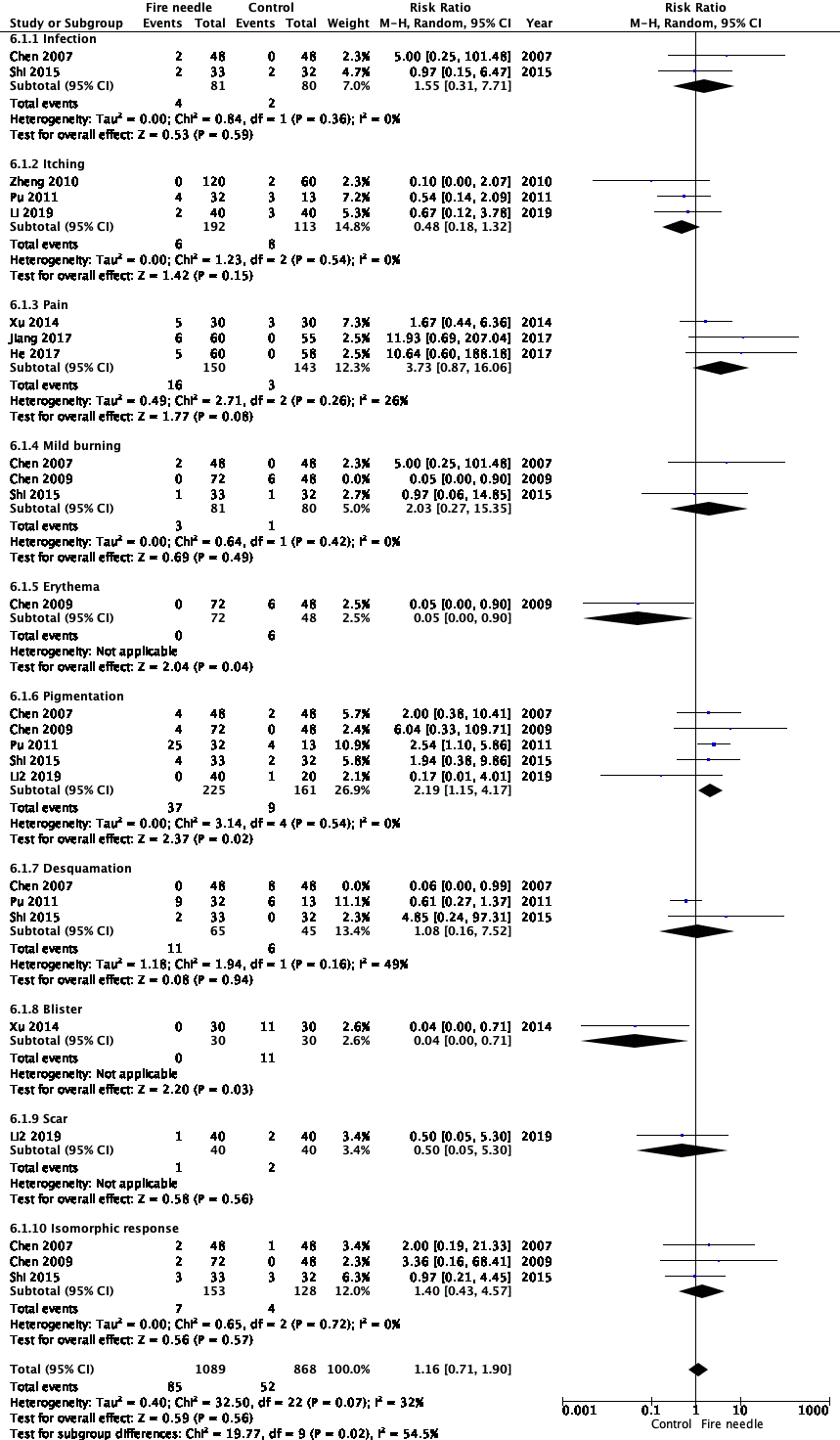


Control groups: tretinoin, immunomodulator, multi-drug therapy, liquid nitrogen freezing, and TCM. TCM: traditional Chinese medicine; CI: indicates confidence interval.

Supplementary Figure S14: Forest plot comparing adverse events between fire needle combined therapies and control groups in a quantitative study on the safety of fire needle therapy for flat warts (sensitivity analysis)


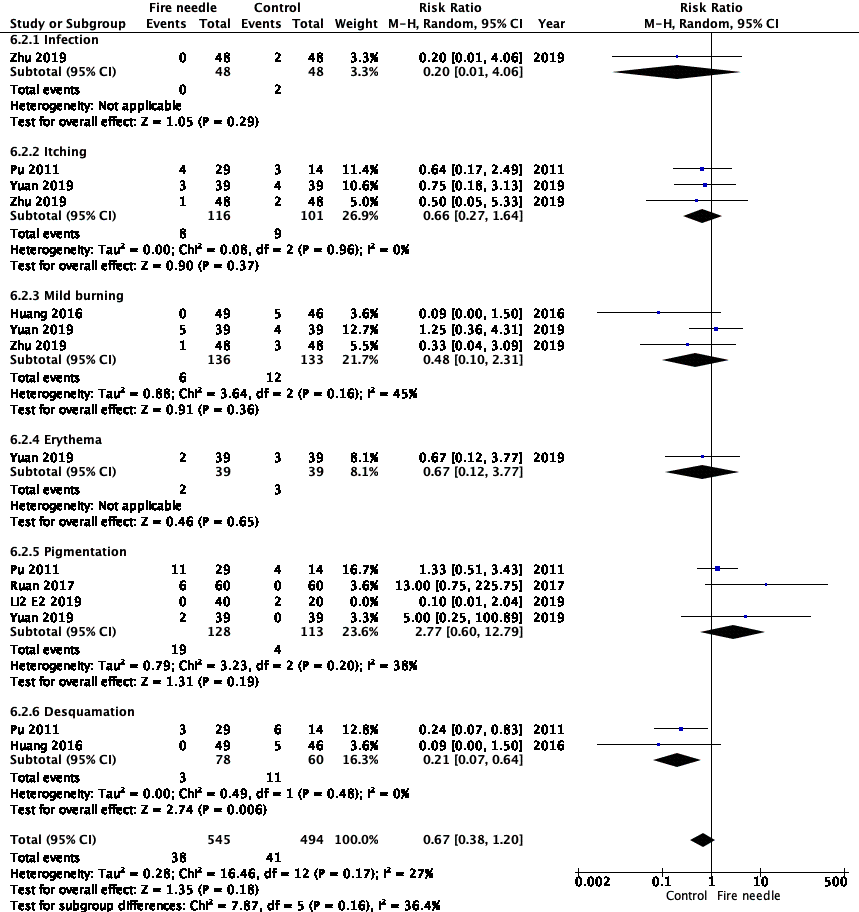


Control groups: imiquimod, multi-drug therapy, TCM, and immunomodulator. TCM: traditional Chinese medicine; CI: indicates confidence interval
